# Plasma Membrane Association by N-Acylation Governs PKG Function in *Toxoplasma gondii*

**DOI:** 10.1128/mBio.00375-17

**Published:** 2017-05-02

**Authors:** Kevin M. Brown, Shaojun Long, L. David Sibley

**Affiliations:** Department of Molecular Microbiology, Washington University School of Medicine, St. Louis, Missouri, USA; Albert Einstein College of Medicine

## Abstract

Cyclic GMP (cGMP)-dependent protein kinase (protein kinase G [PKG]) is essential for microneme secretion, motility, invasion, and egress in apicomplexan parasites, However, the separate roles of two isoforms of the kinase that are expressed by some apicomplexans remain uncertain. Despite having identical regulatory and catalytic domains, PKG^I^ is plasma membrane associated whereas PKG^II^ is cytosolic in *Toxoplasma gondii*. To determine whether these isoforms are functionally distinct or redundant, we developed an auxin-inducible degron (AID) tagging system for conditional protein depletion in *T. gondii*. By combining AID regulation with genome editing strategies, we determined that PKG^I^ is necessary and fully sufficient for PKG-dependent cellular processes. Conversely, PKG^II^ is functionally insufficient and dispensable in the presence of PKG^I^. The difference in functionality mapped to the first 15 residues of PKG^I^, containing a myristoylated Gly residue at position 2 that is critical for membrane association and PKG function. Collectively, we have identified a novel requirement for cGMP signaling at the plasma membrane and developed a new system for examining essential proteins in *T. gondii*.

## IMPORTANCE

*Toxoplasma gondii* is an obligate intracellular apicomplexan parasite and important clinical and veterinary pathogen that causes toxoplasmosis. Since apicomplexans can only propagate within host cells, efficient invasion is critically important for their life cycles. Previous studies using chemical genetics demonstrated that cyclic GMP signaling through protein kinase G (PKG)-controlled invasion by apicomplexan parasites. However, these studies did not resolve functional differences between two compartmentalized isoforms of the kinase. Here we developed a conditional protein regulation tool to interrogate PKG isoforms in *T. gondii*. We found that the cytosolic PKG isoform was largely insufficient and dispensable. In contrast, the plasma membrane-associated isoform was necessary and fully sufficient for PKG function. Our studies identify the plasma membrane as a key location for PKG activity and provide a broadly applicable system for examining essential proteins in *T. gondii*.

## INTRODUCTION

*Apicomplexa* is a large phylum of protozoan parasites that includes several agents of infectious diseases in humans and animals (1). Most apicomplexans replicate exclusively within host cells but also require motile extracellular forms for active transmission between host cells (2). Egress, extracellular migration, and invasion require gliding motility in *Toxoplasma gondii*, a substrate-based form of motility that is unique to members of the phylum *Apicomplexa* (3). To initiate motility, microneme vesicles release membrane-spanning adhesins onto the parasite’s apical surface, where they can bind to extracellular substrates (e.g., host cells, matrix) (4). Next, the cytosolic tails of adhesins engage the actomyosin motor through an adapter called the glideosome-associated connector (5). Rearward motoring of immobilized adhesins by the motor to the posterior pole propels the parasite forward across tissues or into cells (2). Motility in *T. gondii* is paramount to successful invasion and egress; therefore, microneme secretion must be tightly regulated.

Microneme secretion is controlled by two key signaling pathways, calcium (Ca^2+^) and cyclic GMP (cGMP), which direct calcium-dependent protein kinases (CDPKs) (6) and protein kinase G (PKG) (7, 8), respectively, to transduce their respective signals through substrate phosphorylation. Phosphoproteomic studies have identified substrates for CDPK1 in *T. gondii* (9) and PKG in *Plasmodium* spp. (10, 11), yet it is still unclear which phosphorylation events are required for the control of microneme secretion. Interestingly, Ca^2+^ and cGMP may cooperate but also regulate specific steps in microneme secretion. In *T. gondii*, serum albumin stimulates PKG-dependent microneme secretion by elevating cGMP but not Ca^2+^ (12). However, compounds that elevate Ca^2+^ also stimulate microneme secretion (13–15) but only when they receive a second signal such as serum albumin (12). Moreover, elevated calcium cannot overcome PKG inhibition (7), suggesting that PKG may control a final step in this process and act as the master regulator.

Apicomplexan parasites encode a single *PKG* gene (16) that is refractory to deletion in *T. gondii* (17). Selective inhibition of apicomplexan PKG kinase activity is lethal (16–19), supporting an essential role. Despite having a single gene, several genera of tissue cyst-forming coccidian parasites, including *Toxoplasma*, *Hammondia*, *Neospora*, and *Eimeria* species, express two isoforms of PKG that localize to the plasma membrane (PKG^I^) and cytosol (PKG^II^), respectively (16, 17, 19). In *T. gondii*, PKG^I^ (residues 1 to 994) harbors an N-terminal dual-acylation motif that promotes stable association with the plasma membrane, whereas PKG^II^ (residues 103 to 994), which is initiated from a second downstream methionine, lacks this motif and remains cytosolic (19). *PKG* can only be deleted in *T. gondii* in the presence of a second copy of the gene, including those that encode nonacylated mutant proteins (17), although this result might be due to overexpression. Thus, whether PKG^I^ and PKG^II^ are functionally distinct or redundant is currently unknown.

*T. gondii* is equipped with excellent genetic tools, including clustered regularly interspaced short palindromic repeat (CRISPR)/Cas9 genome editing (20, 21). A recent genome-wide CRISPR study performed with *T. gondii* demonstrated that 40% of the 8,158 genes surveyed have a fitness defect *in vitro*, with perhaps only 10% of these being essential (*PKG* included), and identified ~200 hypothetical fitness-conferring genes conserved only in apicomplexans (22). Since the vast majority of these genes have yet to be studied and are likely to be refractory to deletion, conditional genetic technologies are imperative for functional studies. To facilitate the study of essential genes, we adapted the auxin-inducible degron (AID) system that has been shown to degrade proteins of interest in other eukaryotes (23). We utilized a mini-AID (mAID) tagging system for conditional protein depletion that enabled us to resolve functional differences between PKG isoforms in *T. gondii*. This study also highlights a broadly applicable tool for functional analysis of essential proteins in *T. gondii*.

## RESULTS

### Generation of an AID system for conditional protein depletion in *T. gondii.*

**FIG 1:**
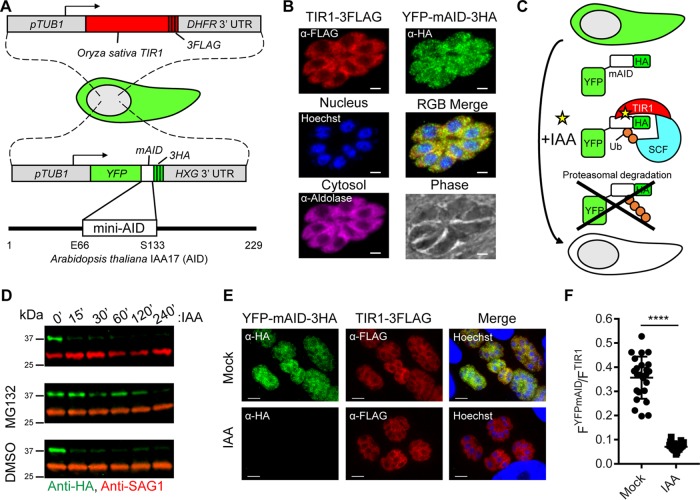
Generation of an AID system in *T. gondii*. (A) Schematic representation of *T. gondii* engineered to coexpress the auxin receptor TIR1 from *Oryza sativa* and YFP fused to mAID from *Arabidopsis thaliana*. (B) Coexpression of TIR1-3FLAG (red) and YFP-mAID-3HA (green) in *T. gondii* determined by IF microscopy. Aldolase (magenta) and Hoechst dye (blue) highlight the cytosol and nuclei, respectively. Scale bars, 2 µm. (C) Schematic representation of conditional YFP-mAID-3HA depletion. Ub, ubiquitin; SCF, Skp-1, Cullin, F-box (TIR1)-containing complex. (D) Western blot assay of lysed YFP-mAID-3HA parasites, probed with antibodies recognizing HA and SAG1. Parasites were treated with 500 µM IAA or the vehicle (EtOH) for up to 240 min in the presence of 50 µM MG132 or the vehicle (DMSO). Data are from a single experiment of two or more experiments with the same outcome. (E) Coexpression of YFP-mAID-3HA (green) and TIR1-3FLAG (red) following treatment with 500 µM IAA or the vehicle (EtOH) for 4 h determined by IF microscopy with the antibodies indicated. Scale bars, 5 µm. (F) Ratiometric quantification of YFP-mAID-3HA to TIR1-3FLAG IF microscopy per vacuole. Mean values of individual vacuoles (EtOH, *n* = 24; IAA, *n* = 23) from two experiments ± the standard deviation, ****, *P* < 0.0001 (unpaired two-tailed Student *t* test).

To rapidly deplete PKG isoforms in *T. gondii*, we developed an auxin-based system for conditional and specific protein depletion (Fig. 1A). We stably expressed a codon-optimized TIR1-3FLAG construct in a *T. gondii* RH line that lacks Ku80 (ku80^KO^) (Fig. 1B). To validate this system, we introduced a yellow fluorescent protein (YFP)-mAID-3HA reporter into TIR1-3FLAG parasites to selectively target this protein for proteasomal degradation (Fig. 1B and C). Addition of auxin promoted rapid depletion of YFP-mAID-3HA, but not the control protein SAG1, within 15 min of treatment (Fig. 1D, top). Pretreatment of parasites with a proteasome inhibitor promoted YFP-mAID-3HA stabilization, confirming the role of the proteasome in YFP-mAID-3HA knockdown (Fig. 1D, middle). Efficient knockdown of YFP-mAID-3HA was independently confirmed and quantified by immunofluorescence (IF) microscopy (Fig. 1E and F).

To adapt the degradation system for biologically important targets, we developed an efficient tagging strategy to incorporate the auxin degron into essential genes. *CDPK1* was selected because it was previously determined to be essential with a transcriptional knockdown system (6). Using a recently described system for efficient CRIPSR/Cas9-mediated gene editing (20), we generated a tagged *CDPK1*-*mAID-3HA* gene in TIR1-3FLAG parasites (see Fig. S1A and B in the supplemental material). CDPK1-mAID-3HA was fully responsive to auxin treatment on the basis of IF microscopy (see Fig. S1C). Auxin-induced depletion of CDPK1-mAID-3HA, but not the control protein aldolase, occurred within minutes of treatment, as shown by Western blotting (see Fig. S1D and E). Degradation of CDPK1-mAID-3HA blocked plaque formation, a measure of the complete lytic cycle, on host cell monolayers (see Fig. S1F), validating the mAID system for the study of essential proteins in *T. gondii*.

10.1128/mBio.00375-17.3 FIG S1  mAID tagging of TIR1-expressing *T. gondii* parasites. (A) CRISPR strategy for tagging of essential genes (e.g., *CDPK1*) with *mAID-3HA* in TIR1-3FLAG parasites. CRISPR DSB, targeted Cas9 double-stranded break. HR, homologous recombination. (B) DNA electrophoretogram of diagnostic PCRs from genomic DNA showing 3′ integration of *mAID-3HA* into *CDPK1*. The genomic loci acting as templates for PCR1 and PCR2 amplicons are shown in panel A. WT (wild type) refers to the TIR1-3FLAG parent. Tag, CDPK1-mAID-3HA parasites. (C) Expression of CDPK1-mAID-3HA (green) and responsiveness to auxin in CDPK1-mAID-3HA parasites determined by IF microscopy following 4 h of treatment with 500 µM IAA or the vehicle (EtOH). Scale bars = 2 µm. (D) Western blot assay of lysed CDPK1-mAID-3HA parasites probed with antibodies recognizing HA and aldolase. Parasites were treated with 500 µM IAA or the vehicle (EtOH). (E) Western blot assay of lysed CDPK1-mAID-3HA parasites probed with antibodies recognizing HA and aldolase. Parasites were treated for 4 h with the concentrations of IAA or the vehicle (EtOH) indicated. (F) Plaque formation by CDPK1-mAID-3HA parasites grown in the presence of 500 µM IAA or the vehicle (EtOH) for 6 days. Shown is the mean number of plaques formed per well (EtOH, *n* = 6; IAA, *n* = 6) from two repeats ± the standard deviation. ****, *P* < 0.0001 (unpaired two-tailed Student *t* test). Panels C to E show data from single experiments of two or more experiments with the same outcomes. Download FIG S1, TIF file, 1.3 MB. Copyright © 2017 Brown et al. 2017 Brown et al. This content is distributed under the terms of the Creative Commons Attribution 4.0 International license.

### Conditional depletion of the PKG^I^ and PKG^II^ isoforms confirms their essentiality in *T. gondii.*

**FIG 2:**
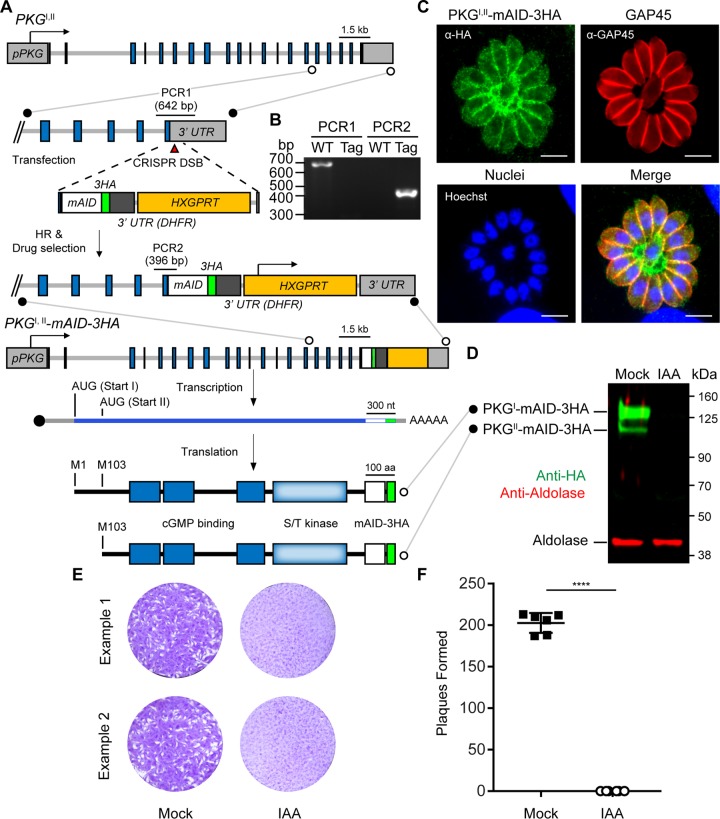
Fusion of *mAID* to PKG^I,II^ in TIR1 parasites allows the simultaneous depletion of PKG^I^ and PKG^II^ isoforms. (A) Strategy for tagging of PKG^I,II^ with *mAID* in TIR1-3FLAG parasites and depiction of the two protein isoforms produced from the PKG^I,II^-*mAID*-*3HA* transcript. (B) DNA electrophoretogram of diagnostic PCRs from genomic DNA showing 3′ integration of *mAID-3HA* into PKG^I,II^. The genomic loci acting as templates for PCR1 and PCR2 amplicons are shown in panel A. Lanes: WT (wild type), TIR1-3FLAG parent; Tag, PKG^I,II^-mAID-3HA parasites. (C) Coexpression of PKG^I^-mAID-3HA and PKG^II^-mAID-3HA (both green) in PKG^I,II^-mAID-3HA parasites determined by IF microscopy. GAP45 (red) is a marker for the parasite plasma membrane. Scale bars = 5 µm. (D) Western blot assay of lysed PKG^I,II^-mAID-3HA parasites probed with antibodies recognizing HA and aldolase. Parasites were treated with 500 µM IAA or the vehicle (EtOH) for 4 h prior to lysis. (E, F) Plaque formation by PKG^I,II^-mAID-3HA parasites treated with 500 µM IAA or the vehicle (EtOH) for 8 days (E) and mean number of plaques formed per well (mock, *n* = 6; IAA, *n* = 6) ± the standard deviation (F). ****, *P* < 0.0001 (unpaired two-tailed Student *t* test). Panels D to F each show data from a single example from three experiments with the same outcome.

To study the function of the PKG isoforms in *T. gondii*, we utilized the mAID system to tag the endogenous locus. Addition of this fusion to the C terminus resulted in the production of both isoforms fused to mAID-3HA (Fig. 2A and B). Previous work has established that PKG^I^ possesses an N-terminal dual-acylation motif that targets it to the plasma membrane, whereas PKG^II^, lacking this motif, remains cytosolic (19). Consistent with this finding, the combined PKG isoform localizations were evident at both the plasma membrane and the cytoplasm (Fig. 2C). Additionally, both mAID-3HA-tagged PKG isoforms were detectable in parasite lysates by Western blotting and both were depleted by auxin treatment for 4 h (Fig. 2D). Depletion of PKG^I,II^-mAID-3HA completely blocked plaque formation on host monolayers (Fig. 2E and F) but did not affect parasite replication (Fig. S2), suggesting a role in motility.

10.1128/mBio.00375-17.4 FIG S2  PKG^I,II^-mAID-3HA knockdown does not affect parasite replication. (A to C) Replication of PKG^I,II^-mAID-3HA parasites following 24 h of treatment with 500 µM IAA or the vehicle (EtOH). Coexpression of PKG^I^-mAID-3HA, PKG^II^-mAID-3HA (both green), and GAP45 (red) in PKG^I,II^-mAID-3HA parasites assessed by IF microscopy (A). Scale bars = 5 µm. Shown are mean percentages of vacuoles containing the number of parasites indicated ± the standard deviation. ns, not significant (two-way analysis of variance) (B). Shown are the mean numbers of parasites per vacuole ± the standard deviation. ns, not significant (unpaired two-tailed Student *t* test) (C). The data are averages of two experiments where, in each experiment, 20 image fields containing approximately 10 to 20 vacuoles were analyzed per treatment condition. Download FIG S2, TIF file, 1.6 MB. Copyright © 2017 Brown et al. 2017 Brown et al. This content is distributed under the terms of the Creative Commons Attribution 4.0 International license.

### Genetic complementation reveals essentiality of PKG^I^ and dispensability of PKG^II^.

**FIG 3:**
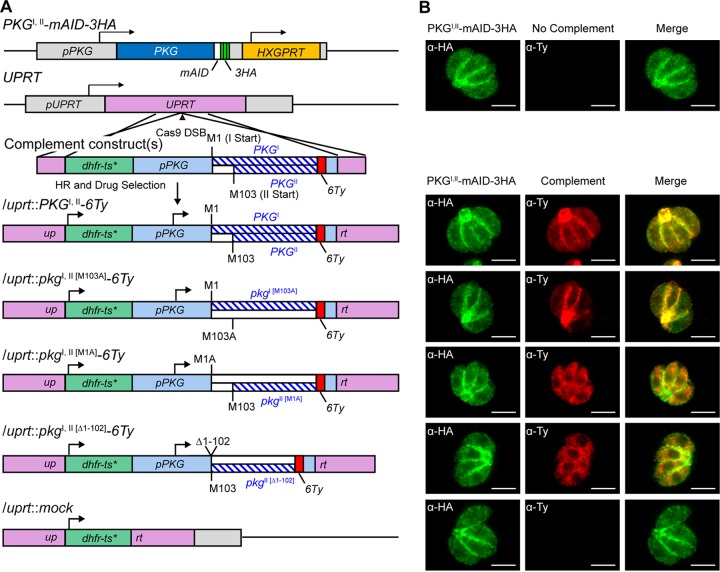
Genetic complementation of strain PKG^I,II^-mAID-3HA. (A) Schematic of the CRISPR/Cas9 strategy used to insert a second copy of *PKG* or mutant isoforms of *pkg* into the *UPRT* locus of PKG^I,II^-mAID-3HA parasites. dhfr-ts*, Pyr^r^ allele. (B) Coexpression of PKG^I,II^-mAID-3HA (both green) and PKG^I,II^-6Ty (red) constructs assessed by IF microscopy. Scale bars = 5 µm.

**FIG 4:**
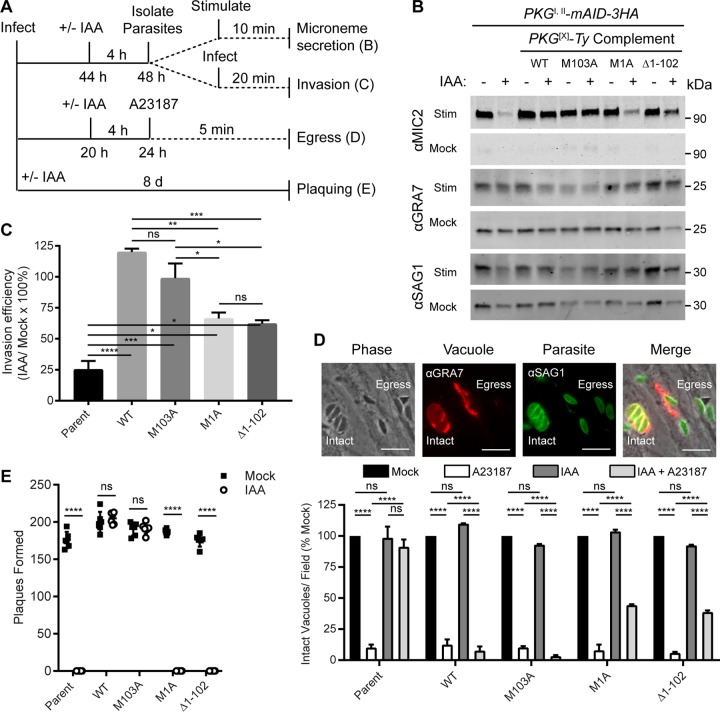
Functional analysis of PKG isoforms. (A) Flow chart showing the experimental design used to test selected PKG-dependent cellular processes. (B) Western blot assay of parasite ESA fractions probed with antibodies recognizing MIC2 (microneme secretion), GRA7 (dense granule secretion), and SAG1 (surface protein shedding). Parasites were treated with 500 µM IAA or the vehicle (EtOH) for 4 h to deplete PKG^I,II^-mAID-3HA and then stimulated with BSA-EtOH or buffer alone prior to ESA collection. (C) Invasion of HFF monolayers following treatment with 500 µM IAA or the vehicle (EtOH) for 4 h to deplete PKG^I,II^-mAID-3HA. Invasion efficiency was calculated as the percentage of the total number of parasites that invaded each host cell in the IAA and mock treatments. Shown are mean values from three experiments each consisting of five replicates per sample and 16 image fields per replicate ± the standard error of the mean. Adjusted *P* values: *, <0.05; **, <0.01; ***, <0.001; ****, <0.0001; ns, not significant (one-way analysis of variance with Tukey’s multiple-comparison test). (D) Egress from HFF monolayers as determined by IF microscopy. Parasites were grown in HFFs for 20 h and treated with 500 µM IAA or the vehicle (EtOH) for 4 h to deplete PKG^I,II^-mAID-3HA and then treated with 4 µM calcium ionophore A23187 for 5 min or left untreated. The micrographs at the top illustrate the difference in intact vacuoles. SAG1 (green) parasites remain tightly clustered and are surrounded by the vacuolar membrane, detected with GRA7 (red), versus those that have egressed and where the parasites are scattered outside the vacuole (marked egress). Scale bars = 10 µm. Mean numbers (from two experiments) of intact vacuoles per field as a percentage of the mock treatment for each strain ± the standard error of the mean are shown. In each experiment, 10 fields per treatment per strain were analyzed. Adjusted *P* value: ****, <0.0001; ns, not significant (two-way analysis of variance with Tukey’s multiple-comparison test). WT, wild type. (E) Plaque formation by parasites treated with 500 µM IAA or the vehicle (EtOH) for 8 days. Shown is the mean number of plaques formed per well (EtOH, *n* = 6; IAA, *n* = 6) ± the standard deviation from a single experiment of three experiments with the same outcome. ****, *P* < 0.0001 (multiple unpaired two-tailed Student *t* test).

To explore the roles of different isoforms, we inserted the wild-type and isoform-specific versions of epitope-tagged PKG (Ty or 6Ty) driven by the endogenous *PKG* promoter into the *UPRT* loci of PKG^I,II^-mAID-3HA parasites (Fig. 3A; Fig. S3A and B) by an established CRISPR knock-in approach (20). Included in this set of lines was a wild-type complement (PKG^I,II^), a complement expressing only PKG^I^ [PKG^I(M103A)^], and two lines that only express PKG^II^ [PKG^II(M1A)^, PKG^II(Δ1-102)^], as well as a mock-complement line (vector only) (Fig. 3A). Correct localization was confirmed by IF microscopy (Fig. 3B), where the wild-type strain showed a mixed pattern of peripheral membrane staining, as well as a residual body with diffuse cytoplasmic staining (Fig. 3B, bottom, top row). The peripheral membrane staining was preserved in the PKG^I^ [PKG^I(M103A)^]-expressing line (Fig. 3B, bottom, second row), while both lines that express only PKG^II^ showed diffuse cytoplasmic staining (Fig. 3B, bottom, third and fourth rows). The two PKG isoforms were clearly distinguished by Western blotting on the basis of differences in molecular weight (Fig. S3C). To examine the roles of the two isoforms in PKG-dependent processes, we compared PKG^I,II^-mAID-3HA parental and complemented parasites for microneme secretion, invasion, and egress by using established assays (Fig. 4A). The efficiency of auxin-induced degradation was crucial for allowing the depletion of wild-type PKG^I,II^-AID-3HA during 4 h of treatment with auxin, thereby revealing the phenotypes of the various complementing forms in these short-term assays (Fig. 4A).

10.1128/mBio.00375-17.5 FIG S3  Supplement to PKG^I,II^-mAID-3HA complementation. (A) Schematic of *UPRT* locus disrupted with a dhfr-ts* PKG^I,II^*-Ty* complementation construct showing template regions for diagnostic PCRs 1 to 3. dhfr-ts*, Pyr^r^ allele. (B) Agarose gel of diagnostic PCRs 1 to 3 (described in panel A) demonstrating genomic integration of complementation constructs. (C) Western blot assay of parasite lysates probed with antibodies recognizing HA (green) and Ty (red). WT, wild type. Download FIG S3, TIF file, 0.8 MB. Copyright © 2017 Brown et al. 2017 Brown et al. This content is distributed under the terms of the Creative Commons Attribution 4.0 International license.

Auxin-induced PKG depletion specifically blocked microneme secretion induced by ethanol (EtOH) and serum albumin, while the release of dense granules (GRA7) and surface proteins (SAG1) was unaffected (Fig. 4B, lanes 1 to 2). Importantly, both wild-type PKG^I,II^ and PKG^I^-specific constructs fully rescued microneme secretion following PKG^I,II^-mAID-3HA depletion (Fig. 4B, lanes 3 to 6). Interestingly, PKG^II^-specific constructs only partially supported microneme secretion following PKG^I,II^-mAID-3HA depletion (Fig. 4B, lanes 7 to 10), suggesting that microneme-dependent processes would also be affected. Similarly, depletion of PKG^I,II^-mAID-3HA caused an ~75% reduction in invasion efficiency (Fig. 4C). Complementation with wild-type PKG^I,II^ and PKG^I^-specific constructs fully rescued invasion, whereas only partial rescue was obtained with PKG^II^-specific constructs (Fig. 4C). Likewise, wild-type PKG^I,II^ and PKG^I^-specific constructs, but not PKG^II^-specific constructs, fully supported calcium ionophore-induced egress from host cell monolayers following PKG-mAID-3HA depletion (Fig. 4D). Given the partial sufficiency of PKG^II^ to function in microneme secretion and related processes, we asked whether PKG^II^ alone could support parasite growth and fitness over the course of several days. Although PKG^II^-complemented lines could partially support microneme secretion, invasion, and egress, they were insufficient at supporting plaque formation (Fig. 4E). Conversely, parasites complemented with wild-type PKG^I,II^ or PKG^I^-specific constructs were fully capable of forming plaques on human foreskin fibroblast (HFF) monolayers upon PKG^I,II^-mAID-3HA depletion (Fig. 4E).

**FIG 5:**
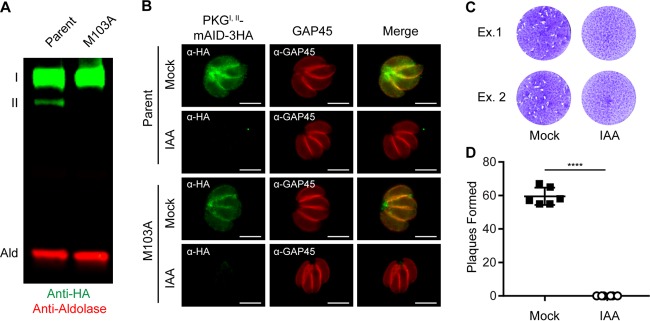
PKG^II^ is dispensable in the presence of PKG^I^. (A) Western blot assay of parasite lysates probed with antibodies recognizing HA (green) and aldolase (red). (B) Coexpression of PKG^I,II^-mAID-3HA (both green) and GAP45 (red) in parasites assessed by IF microscopy. Scale bars = 5 µm. (C, D) Plaque formation by parasites treated with 500 µM IAA or the vehicle (EtOH) for 8 days (C) and mean number of plaques formed per well (mock, *n* = 6; IAA, *n* = 6) ± the standard deviation (D). ****, *P* < 0.0001 (unpaired two-tailed Student *t* test). Panels B to D show data from single experiments of sets of three experiments with the same outcome. The micrograph rows in panel B correspond to the adjacent schematic in panel A.

To confirm that PKG^II^ is dispensable, we developed a markerless genome editing strategy to introduce an M103A mutation into the endogenous *PKG-mAID-3HA* gene, thereby preventing PKG^II^ translation (Fig. S4A). We cotransfected parasites with a Cas9-green fluorescent protein (GFP)/guide RNA plasmid that cuts 6 bp upstream from the M103 codon and a Cas9-shielded homology donor amplicon corresponding to exon 3 (252 bp) containing the M103A mutation. Following transfection, parasites transiently expressing Cas9-GFP were sorted with a fluorescence-activated cell sorter (FACS) (Fig. S4B) and expanded on host cell monolayers (Fig. S4C). We hypothesized that parasites that expressed Cas9 would only survive a double-stranded break in *PKG* if it were repaired with the donor M103A template. Single clones were isolated from the FACS-sorted population by limiting dilution, and a 1-kb fragment surrounding the *PKG* M103 allele was amplified by PCR and sequenced by the Sanger method. The M103A and shielding mutations were evident in a transfected clone but not the parental line, confirming successful markerless editing (Fig. S4D). We confirmed loss of PKG^II^ at the protein level by Western blotting, demonstrating that this line only expresses PKG^I^ (Fig. 5A). To rule out compensatory mutations during genome editing, we depleted PKG^I^ (Fig. 5B) and found that parasites were incapable of growth (Fig. 5C and D). Taken together, these data reveal that PKG^II^ is dispensable, while PKG^I^ is essential, for growth on host cell monolayers.

10.1128/mBio.00375-17.6 FIG S4  Disruption of endogenous *PKG*^II^ by markerless genome editing. (A) Schematic illustration of a markerless CRISPR/Cas9 genome editing strategy used to introduce an M103A substitution mutation into PKG^I,II^*-mAID-3HA* to disrupt the *PKG*^II^ translation start methionine codon. (B) FACS plot showing the gate used to sort Cas9-GFP^+^ parasites following transfection of PKG^I,II^-mAID-3HA parasites with pSAG1:CAS9-GFP, U6:sgPKG(M103), and a Cas9-shielded PKG exon 3 (M103A) donor amplicon. (C) Live-cell fluorescence micrograph showing FACS-sorted parasites transiently expressing nuclear Cas9-GFP (green) expanding on an HFF monolayer prior to cloning. (D) Sanger sequencing chromatograms of purified PCR amplicons from PKG^I,II^-mAID-3HA (parent) and PKG^I(M103A)^-mAID-3HA (M103A) parasites confirming the PKG genome edit. Download FIG S4, TIF file, 1.3 MB. Copyright © 2017 Brown et al. 2017 Brown et al. This content is distributed under the terms of the Creative Commons Attribution 4.0 International license.

### Plasma membrane association is critical for PKG function in *T. gondii.*

**FIG 6:**
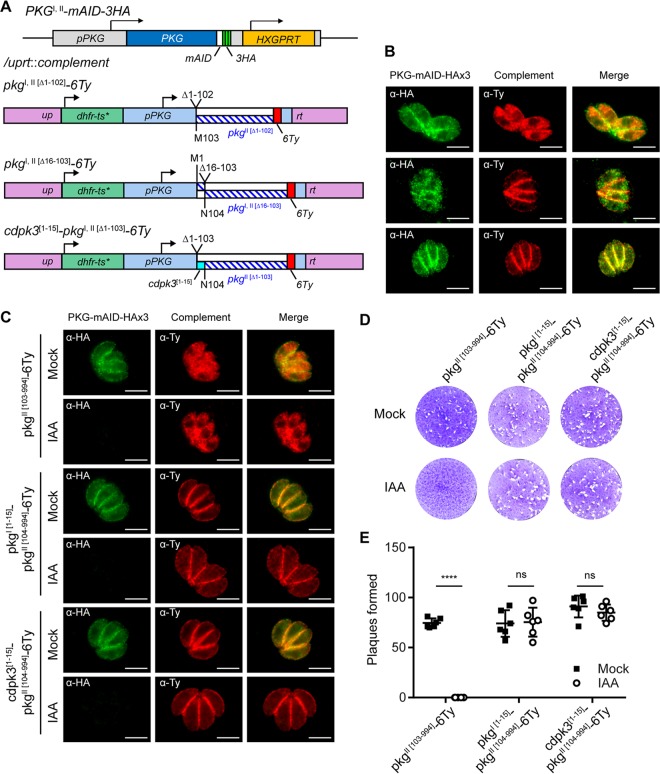
Plasma membrane localization functionally distinguishes PKG^I^ from PKG^II^. (A) Schematic of the CRISPR/Cas9 strategy used to insert a second copy of *PKG* or mutant isoforms of *pkg* into the *UPRT* locus of PKG^I,II^-mAID-3HA parasites. dhfr-ts*, Pyr^r^ allele. (B) Coexpression of PKG^I,II^-mAID-3HA (both green) and PKG-6Ty complementation constructs (red) assessed by IF microscopy. Scale bars = 5 µm. The micrograph rows correspond to the adjacent schematic in panel A. (C) Coexpression of PKG^I,II^-mAID-3HA (both green) and PKG-6Ty complementation constructs (red) following 4 h of treatment with 500 µM IAA or the vehicle (EtOH) assessed by IF microscopy. Scale bars = 5 µm. (D, E) Plaque formation by parasites treated with 500 µM IAA or the vehicle (EtOH) for 8 days (D) and mean number of plaques formed per well (mock, *n* = 6; IAA, *n* = 6) ± the standard deviation from two experiments (E). ****, *P* < 0.0001; ns, not significant (unpaired two-tailed Student *t* test). Panels B and C show data from one of at least two experiments with the same outcome.

The observation that PKG^II^ was unable to fully complement microneme secretion and sustain parasite growth was intriguing, given that the isoform-specific complementation constructs showed similar levels of protein expression (Fig. S3C). Other than expression, the only known difference between the PKG^I^ and PKG^II^ isoforms is the presence of an N-terminal extension that contains a dual myristoylation and palmitoylation motif that is required for the plasma membrane association of PKG^I^ (19). We hypothesized that in *T. gondii*, PKG activity at the plasma membrane is critically important for PKG function and parasite fitness. To test this hypothesis, we first needed to define the PKG^I^ motif that could direct a soluble protein to the plasma membrane. We generated an mNeon-6Ty fusion with the first 15 amino acids (aa) of PKG^I^ that was able to direct this fluorescent reporter to the plasma membrane (Fig. S5A to C). Plasma membrane association is likely to require glycine myristoylation at position 2 of PKG^I^ since a G2A mutant version of this peptide fused to mNeon-6Ty remained cytosolic (Fig. S5A to C). Likewise, we found that the first 15 aa of PKG^I^ were sufficient to redirect PKG^II^ from the cytosol to the plasma membrane (Fig. 6A and B, top and middle). To rule out any unforeseen PKG^I^-specific functions that may be present in the 15-aa peptide, we also directed PKG^II^ to the plasma membrane with a similar N-terminal acylated peptide from CDPK3 (24) (Fig. 6A and B, bottom). The expression levels and localization of the ectopic PKG^II^ constructs were independent of the endogenous copy of PKG^I,II^ (Fig. 6C). Using these tools, we asked whether PKG^II^ directed to the plasma membrane by N-terminal PKG^I^ aa 1 to 15 or CDPK3 aa 1 to 15 fusions was sufficient to support parasite growth and fitness following PKG^I,II^-mAID-3HA depletion in a plaque formation assay. We observed that parasites complemented with a cytosolic PKG^II^ construct could not form plaques on host cell monolayers following auxin-induced PKG^I,II^-mAID-3HA depletion (Fig. 6D and E, left column). Surprisingly, parasites complemented with plasma membrane-associated PKG^II^ constructs were fully capable of forming plaques when PKG^I,II^-mAID-3HA was degraded by growth in auxin (Fig. 6D and E, middle and right columns). Therefore, we conclude that in *T. gondii*, PKG localization to the plasma membrane, but not the cytosol, is essential for proper PKG function and parasite viability.

10.1128/mBio.00375-17.7 FIG S5  The PKG^I^ N-terminal peptide (aa 1 to 15) is sufficient for plasma membrane targeting and requires glycine at position 2. (A) Schematic of mNeon-6Ty expression constructs with or without N-terminal PKG^I^ peptide fusions. (B) Live-cell spinning-disc confocal micrographs of parasites stably transfected with mNeon-6Ty expression constructs (green) with or without N-terminal PKG^I^ peptide fusions grown in HFF monolayers. Scale bars = 5 µm. (C) Fixed-cell spinning-disc confocal micrographs of parasites stably transfected with mNeon-6Ty expression constructs with or without N-terminal PKG^I^ peptide fusions assessed by IF microscopy with the antibodies indicated. Scale bars = 5 µm. Panels B and C show data from one of two experiments with the same outcome. The micrograph rows in panels B and C correspond to the adjacent schematic in panel A. Download FIG S5, TIF file, 0.7 MB. Copyright © 2017 Brown et al. 2017 Brown et al. This content is distributed under the terms of the Creative Commons Attribution 4.0 International license.

## DISCUSSION

Chemical genetic studies have demonstrated that PKG activity is required for the lytic life cycles of *T. gondii* and other apicomplexan parasites (16–19, 25). However, technical limitations of these methods have precluded the assignment of specific functions to membrane and soluble forms of PKG in parasites that express two isoforms. To define the functional contributions of each PKG isoform, we developed an AID tagging system that allowed rapid and robust conditional protein depletion in *T. gondii*. Simultaneous knockdown of both PKG isoforms was lethal, confirming their essentiality. Using a highly efficient CRISPR/Cas9 system for genome editing, combined with the AID degradation system, we found that plasma membrane-associated PKG^I^ was necessary and sufficient, whereas cytosolic PKG^II^ was largely insufficient and dispensable. Importantly, we were able to impart sufficiency to cytosolic PKG^II^ by artificially directing it to the plasma membrane with N-terminal PKG^I^ or CDPK3 peptide fusions. The combination of CRISPR/Cas9-mediated editing with mAID conditional protein regulation provides a powerful system for interrogation of the roles of essential proteins, including closely related isoforms of PKG.

Much of what is known about PKG function in apicomplexans was inferred from the effects of the PKG inhibitors, including the trisubstituted pyrrole inhibitor (compound 1) (16) and the imidazopyridine inhibitor (compound 2) (26). PKG was validated as a target by using gatekeeper mutants that were resistant to compound 1 and 2 inhibition (27). However, both compounds 1 and 2 have activity on other kinases, including CDPK1, which controls functions similar to those of PKG (26). Additional PKG-independent effects of compound 1 include induction of tissue cyst development in *T. gondii* (28, 29). Furthermore, gatekeeper sensitization alone may lead to artificial results due to functional compensation, as was recently described for a gatekeeper mutant form of CDPK1 in *P. falciparum* (30). Thus, although PKG appears to be an essential gene in *T. gondii*, systems that allow careful dissection of the roles of two different isoforms have been lacking.

Conditional gene regulation is a powerful approach for understanding gene product function, and several such systems have been implemented in *T. gondii* (reviewed in reference 31). Transcriptional systems are relatively slow since they require natural turnover of existing mRNA and/or protein. Gradual depletion of proteins can lead to artificial phenotypes or mask genuine phenotypes, as has been demonstrated for the yeast helicase gene *MCM4* (23, 32). In contrast, posttranslational systems allow the rapid depletion of gene products (proteins) of interest. For instance, ddFKBP is an intrinsically unstable regulatable protein degron that is stabilized by the compound shield-1 (33). When used previously in *T. gondii*, addition of shield-1 fully stabilized a ddFKBP-YFP reporter within 90 min, whereas removal of shield-1 depleted ddFKBP-YFP to the background level after 320 min (34), representing a substantial improvement in speed over other systems. Unfortunately, the ddFKBP knockdown system requires permanent treatment of shield-1, an expensive compound known to have some toxicity for apicomplexan parasites (35), prompting us to develop an alternative approach.

Unlike the ddFKBP system, the AID system uses an inexpensive and innocuous plant hormone (auxin/indole-3-acetic acid [IAA]) that is applied only when knockdown of an AID fusion is desired (23). To regulate proteins of interest with the AID system, two novel genetic components are needed. First, the cell must express an auxin receptor called transport inhibitor response 1 (TIR1) that combines to form a functional SCF ubiquitin ligase complex (SCF^TIR1^) in the presence of auxin (23). Here we expressed a codon-optimized version of TIR1 in *T. gondii*, as earlier attempts with the *OsTIR1* gene from rice failed. Second, proteins targeted for depletion must be linked to an AID, which may be as small as 68 aa (36). Addition of auxin promotes polyubiquitination of AID-tagged proteins by the SCF^TIR1^ complex, targeting them for proteasomal degradation. The AID system has previously been used in *Plasmodium* spp. to examine the roles of essential proteins (37, 38), prompting us to import the system into *T. gondii*. In our experience, the AID system operates an order of magnitude faster than the ddFKBP system in *T. gondii* (30 min for AID, as shown here, versus 320 min [34]). Furthermore, truncated versions of AID function efficiently with only 43 to 68 aa (36, 39), limiting potential interference caused by bulky fusions. On the basis of these properties, the mAID system provides an efficient ligand-off protein expression tool in *T. gondii* that features unmatched specific protein knockdown speeds with an inexpensive, nontoxic ligand (auxin/IAA).

We applied the mAID system to *PKG* to confirm its essentiality and resolve functional differences between PKG isoforms in *T. gondii*. We found that plasma membrane-associated PKG^I^ was necessary and sufficient for wild-type level function, whereas cytosolic PKG^II^ was largely insufficient and dispensable. PKG^II^ mutants that were artificially targeted to the plasma membrane were also sufficient, revealing a novel plasma membrane association requirement for PKG function in this parasite. These results were unexpected for two reasons. First, most of the PKGs of apicomplexan parasites with sequenced genomes and gene annotations lack the dual-acylation motif required for plasma membrane association and are likely cytosolic (40). However, such cytosolic isoforms of PKG should still have access to the plasma membrane by diffusion and may transiently fulfill a functional requirement for PKG at this interface. In fact, PKG-dependent phosphoproteomic studies performed with *Plasmodium* spp. identified several potential substrates at the plasma membrane (10, 11). Second, a previous study of *T. gondii* showed that endogenous PKG could be deleted in parasites expressing *Eimeria tenella* FLAG-PKG, where the FLAG epitope was thought to disrupt the dual-acylation sequence and promote cytosolic localization, implying that plasma membrane association is not required for PKG function (17). However, since *E. tenella* FLAG-PKG was expressed by the *T. gondii* tubulin promoter (17), it is possible that its overexpression was responsible for compensating for loss of PKG^I^ at the plasma membrane. In our studies with the endogenous promoter, we found that PKG^II^ alleles were not sufficient to rescue function, unless directed to the plasma membrane by an N-terminal fusion carrying an acylation signal.

In addition to *T. gondii*, several other tissue cyst-forming coccidian parasites, including *Hammondia*, *Neospora*, and *Eimeria* species, also carry an additional isoform of PKG to function at the plasma membrane. The reason why this location is essential in *T. gondii* while a soluble form is sufficient in *Plasmodium* and other genera is uncertain. However, it is possible that by directing the enzyme to the membrane, the overall expression level can be reduced, making substrate engagement more specific and preventing nonproductive phosphorylation of undesirable targets. Consistent with this model, evidence that PKG can compensate for other plasma membrane-directed kinases such as CDPK3 during egress by *T. gondii* (41) suggests a necessity for tight control or spatial restriction of PKG activity. It is also unclear why species that retain both plasma membrane and cytosolic forms retain the latter form, as our functional studies performed here suggest that the cytosolic form is dispensable. Our findings may reflect a vestigial function for PKG^II^ or alternatively suggest a role *in vivo* that has not been tested in the present study. Collectively, our studies demonstrate the power of combining CRISPR/Cas9 genome engineering with conditional protein regulation to determine gene function and essentiality. Our studies identify the plasma membrane as the central platform for cGMP effector signaling in *T. gondii*, which should greatly facilitate the identification of currently unknown substrates of PKG that are required for microneme secretion.

## MATERIALS AND METHODS

### Parasite strains and growth conditions.

Proposed genetic nomenclature guidelines for *T. gondii* genes, plasmids, and transgenic lines are shown in Text S2. Parental *T. gondii* strain ku80^KO^ (genotype RHΔ*hxgprt*Δ*ku80* [42]) and transgenic lines and associated plasmids are listed in Table S3. Parasites were cultivated in HFF monolayers in D3 medium (Dulbecco’s modified Eagle’s medium [Invitrogen] supplemented with 3% HyClone fetal bovine serum [GE Healthcare Life Sciences], 10 μg/ml gentamicin, 10 mM glutamine [Thermo Fisher Scientific]). All strains and host cell lines were determined to be mycoplasma negative with the e-Myco plus kit (Intron Biotechnology).

### Chemicals and antibodies.

IAA, MG-132, bovine serum albumin (BSA), A23187, and all other chemicals were purchased from Sigma-Aldrich unless indicated otherwise. Rat anti-FLAG (BioLegend), mouse anti-hemagglutinin (HA) (BioLegend), and rabbit anti-HA (Thermo Fisher Scientific) antibodies were purchased commercially. Mouse anti-Ty (43), rabbit anti-aldolase (44), mouse anti-MIC2 (45), and rabbit anti-GRA7 (46) antibodies were raised in house. Rabbit anti-GAP45 antibody (47) was provided by Dominique Soldati-Favre (University of Geneva), and rabbit anti-SAG1 antibody (48) was provided by John Boothroyd (Stanford University). Goat secondary antibodies conjugated to infrared (IR) and Alexa Fluor dyes were purchased from LiCor and Thermo Fisher Scientific, respectively.

### Plasmid construction.

The plasmids and primers used in this study are listed in Tables S1 and S2, respectively. Detailed plasmid construction information is presented in Text S1. Synthetic DNA fragments (gBlocks) and primers were purchased from Integrated DNA Technologies, Inc. Reagents from New England Biolabs Inc. were used for PCR (Q5 polymerase), restriction digestions (various restriction enzymes), all ligations (T4 DNA ligase, Gibson assembly cloning kit), and mutagenesis (Q5 site-directed mutagenesis kit) in accordance with the manufacturer’s instructions. Plasmid sequences were confirmed by Sanger sequencing (Genewiz Inc.).

10.1128/mBio.00375-17.8 TABLE S1  Plasmids used in this study. Download TABLE S1, DOCX file, 0.02 MB. Copyright © 2017 Brown et al. 2017 Brown et al. This content is distributed under the terms of the Creative Commons Attribution 4.0 International license.

10.1128/mBio.00375-17.9 TABLE S2  Primers used in this study. Download TABLE S2, DOCX file, 0.02 MB. Copyright © 2017 Brown et al. 2017 Brown et al. This content is distributed under the terms of the Creative Commons Attribution 4.0 International license.

10.1128/mBio.00375-17.1 TEXT S1  Plasmid construction. Download TEXT S1, DOCX file, 0.02 MB. Copyright © 2017 Brown et al. 2017 Brown et al. This content is distributed under the terms of the Creative Commons Attribution 4.0 International license.

10.1128/mBio.00375-17.2 TEXT S2  Appendix of proposed genetic nomenclature guidelines for *T. gondii*. Download TEXT S2, DOCX file, 0.1 MB. Copyright © 2017 Brown et al. 2017 Brown et al. This content is distributed under the terms of the Creative Commons Attribution 4.0 International license.

10.1128/mBio.00375-17.10 TABLE S3  Strains used in this study. Download TABLE S3, DOCX file, 0.01 MB. Copyright © 2017 Brown et al. 2017 Brown et al. This content is distributed under the terms of the Creative Commons Attribution 4.0 International license.

### Generation of transgenic parasites.

Freshly harvested parasites were transfected with purified plasmid or amplicon DNA by electroporation as previously described (49). Transgenic parasites were selected following DNA transfection by FACS sorting with a FACSAria II (BD Biosciences) on the basis of Cas9-GFP fluorescence or with an appropriate antibiotic. When needed, the antibiotic (concentration) used for drug selection was chloramphenicol (20 µM), mycophenolic acid (25 µg/ml) with xanthine (50 µg/ml), pyrimethamine (3 µM), or 5-fluorodeoxyuracil (10 µM). Stable clones were isolated by limiting dilution.

### Auxin-induced depletion of mAID-tagged proteins.

A stock of 500 mM IAA dissolved in 100% EtOH at 1:1,000 was used to deplete mAID-tagged proteins at a final concentration of 500 µM. Mock treatment consisted of an equivalent volume of 100% EtOH at a final concentration of 0.0789%, wt/vol.

### IF microscopy.

Parasite-infected HFF monolayers grown on glass coverslips, 96-well plates, or coverslip dishes were fixed with 4% formaldehyde, permeabilized with 0.1% Triton X-100, blocked with 5% fetal bovine serum--5% normal goat serum, labeled with primary antibodies, and then washed with phosphate-buffered saline (PBS). Antibody-labeled proteins were fluorescently labeled with Alexa Fluor-conjugated secondary goat antibodies. Nuclei were stained with Hoechst 33342 dye. Standard wide-field images were captured and analyzed with a 63× or 100× oil objective on an Axioskop 2 MOT Plus wide-field fluorescence microscope (Carl Zeiss, Inc.) running AxioVision LE64 software (Carl Zeiss, Inc.). High-throughput imaging and analysis were performed with a Cytation 3 (BioTek) multimode plate imager with a 20× objective running Gen5 software (BioTek). Spinning-disc confocal images of live or immunolabeled cells were captured and analyzed on an AxioObserver Z1 (Carl Zeiss, Inc.) with a 100× oil objective running Zen 2 software (Carl Zeiss, Inc.).

### Western blotting.

Protein samples were diluted 4:1 in 5× Laemmli buffer containing 100 mM dithiothreitol, boiled for 5 min, separated on 4 to 15% Mini-PROTEAN TGX polyacrylamide gels (Bio-Rad Laboratories, Inc.) by SDS-PAGE, and transferred to nitrocellulose membranes. The membranes were blocked with 5% (wt/vol) fat-free milk in PBS and then probed with primary antibodies diluted in blocking buffer containing 0.1% Tween 20. Membranes were washed with PBS--0.1% Tween 20, and antibody-labeled antigens were visualized with IR dye-conjugated secondary antibodies on a LiCor Odyssey imaging system (LI-COR Biosciences).

### Plaque formation.

Freshly harvested parasites were counted, and 200 or 1,000 parasites were added to six-well plates of confluent HFF monolayers in D3 medium. Wells were treated with 500 µM IAA to deplete mAID fusion proteins or the vehicle (EtOH), and plaques were allowed to form for 6 to 8 days, depending on the experiment. Plaque formation was assessed by counting the zones of clearance on EtOH-fixed, crystal violet-stained HFF monolayers.

### Parasite growth.

Freshly harvested parasites were allowed to invade HFF monolayers grown on glass coverslips for 1 h to allow invasion, and then cultures were treated with 500 µM IAA to deplete mAID-3HA fusion proteins or the vehicle (EtOH) and grown for an additional 23 h. Infected monolayers were fixed at 24 h, and the number of parasites per vacuole was determined by IF microscopy. In each experiment, 20 image fields containing approximately 10 to 20 vacuoles were analyzed per treatment condition.

### Microneme secretion.

Parasites grown in HFF monolayers were pretreated with 500 µM IAA to deplete mAID-3HA fusion proteins or the vehicle (EtOH) for 4 h. Parasites were then syringe released, 3 µm filtered, washed, and resuspended in extracellular (EC) buffer (5 mM KCl, 142 mM NaCl, 1 mM MgCl_2_, 1.8 mM CaCl_2_, 5.6 mM d-glucose, 25 mM HEPES, pH 7.4), and 2 × 10^7^ parasites were stimulated with 1% (wt/vol) BSA--1% (vol/vol) EtOH (final concentrations) in EC buffer or in EC buffer alone for 10 min at 37°C in the presence or absence of 500 µM IAA. Following stimulation, parasites were chilled on ice and pelleted at 400 × *g* for 10 min. Excreted/secreted antigen (ESA) fractions were collected and centrifuged once more at 800 × *g* for 10 min. The twice cleared cell-free ESA fractions were subjected to Western blotting to assess microneme secretion. In each experiment, one replicate per treatment per strain was analyzed.

### Parasite invasion.

Parasites grown in HFF monolayers were pretreated with 500 µM IAA to deplete mAID-3HA fusion proteins or the vehicle (EtOH) for 4 h. Parasites were then harvested, resuspended in D3, and used to infect HFF monolayers grown in optically clear 96-well plates (Greiner) for 20 min at 37°C in the presence of 500 µM IAA or the vehicle (EtOH). Invasion was stopped by formaldehyde fixation, extracellular parasites were exclusively labeled with mouse anti-SAG1--Alexa Fluor 594 conjugate, and unbound antibody was removed by washing with PBS. The monolayers were then permeabilized with 0.1% Triton X-100, and all parasites were labeled with mouse anti-SAG1--Alexa Fluor 488 conjugate. Host nuclei were stained with Hoechst 33342 dye. A Cytation 3 multimode plate imager running Gen5 software (BioTek Instruments) was used to image parasites and quantitate the number that invaded each host cell. In each experiment, five replicates per sample and 16 image fields per replicate were analyzed.

### Parasite egress.

Freshly harvested parasites were resuspended in D3 and counted, and 5 × 10^4^ parasites were allowed to invade HFF monolayers grown on glass coverslips for 20 h. Parasites were then pretreated with 500 µM IAA for 4 h to deplete mAID-3HA fusion proteins or the vehicle (EtOH). To stimulate egress, parasites were treated with 4 µM (final concentration) calcium ionophore A23187 or the vehicle for 5 min at 37°C. Egress was stopped by formaldehyde fixation and evaluated by IF microscopy following permeabilization, blocking, and immunolabeling with antibodies against the parasite (SAG1) and parasitophorous vacuole (GRA7). In each experiment, 10 fields per treatment per strain were analyzed.

## References

[1] Seeber F, Steinfelder S 2016 Recent advances in understanding apicomplexan parasites. F1000Res 5:F1000 Faculty Rev-1369. doi:10.12688/f1000research.7924.1. PMC4909106 27347391

[2] Sibley LD 2010 How apicomplexan parasites move in and out of cells. Curr Opin Biotechnol 21:592–598. doi:10.1016/j.copbio.2010.05.009. 20580218 PMC2947570

[3] Heintzelman MB 2015 Gliding motility in apicomplexan parasites. Semin Cell Dev Biol 46:135–142. doi:10.1016/j.semcdb.2015.09.020. 26428297

[4] Carruthers VB, Tomley FM 2008 Microneme proteins in apicomplexans. Subcell Biochem 47:33–45. doi:10.1007/978-0-387-78267-6_2. 18512339 PMC2847500

[5] Jacot D, Tosetti N, Pires I, Stock J, Graindorge A, Hung YF, Han H, Tewari R, Kursula I, Soldati-Favre D 2016 An apicomplexan actin-binding protein serves as a connector and lipid sensor to coordinate motility and invasion. Cell Host Microbe 20:731–743. doi:10.1016/j.chom.2016.10.020. 27978434

[6] Lourido S, Shuman J, Zhang C, Shokat KM, Hui R, Sibley LD 2010 Calcium-dependent protein kinase 1 is an essential regulator of exocytosis in toxoplasma. Nature 465:359–362. doi:10.1038/nature09022. 20485436 PMC2874977

[7] Wiersma HI, Galuska SE, Tomley FM, Sibley LD, Liberator PA, Donald RGK 2004 A role for coccidian cGMP-dependent protein kinase in motility and invasion. Int J Parasitol 34:369–380. doi:10.1016/j.ijpara.2003.11.019. 15003497

[8] Howard BL, Harvey KL, Stewart RJ, Azevedo MF, Crabb BS, Jennings IG, Sanders PR, Manallack DT, Thompson PE, Tonkin CJ, Gilson PR 2015 Identification of potent phosphodiesterase inhibitors that demonstrate cyclic nucleotide-dependent functions in apicomplexan parasites. ACS Chem Biol 10:1145–1154. doi:10.1021/cb501004q. 25555060

[9] Lourido S, Jeschke GR, Turk BE, Sibley LD 2013 Exploiting the unique ATP-binding pocket of toxoplasma calcium-dependent protein kinase 1 to identify its substrates. ACS Chem Biol 8:1155–1162. doi:10.1021/cb400115y. 23530747 PMC3691715

[10] Brochet M, Collins MO, Smith TK, Thompson E, Sebastian S, Volkmann K, Schwach F, Chappell L, Gomes AR, Berriman M, Rayner JC, Baker DA, Choudhary J, Billker O 2014 Phosphoinositide metabolism links cGMP-dependent protein kinase G to essential Ca^2+^ signals at key decision points in the life cycle of malaria parasites. PLoS Biol 12:e1001806. doi:10.1371/journal.pbio.1001806. 24594931 PMC3942320

[11] Alam MM, Solyakov L, Bottrill AR, Flueck C, Siddiqui FA, Singh S, Mistry S, Viskaduraki M, Lee K, Hopp CS, Chitnis CE, Doerig C, Moon RW, Green JL, Holder AA, Baker DA, Tobin AB 2015 Phosphoproteomics reveals malaria parasite protein kinase G as a signalling hub regulating egress and invasion. Nat Commun 6:7285. doi:10.1038/ncomms8285. 26149123 PMC4507021

[12] Brown KM, Lourido S, Sibley LD 2016 Serum albumin stimulates protein kinase G-dependent microneme secretion in Toxoplasma gondii. J Biol Chem 291:9554–9565. doi:10.1074/jbc.M115.700518. 26933037 PMC4850294

[13] Carruthers VB, Sibley LD 1999 Mobilization of intracellular calcium stimulates microneme discharge in *Toxoplasma gondii*. Mol Microbiol 31:421–428. doi:10.1046/j.1365-2958.1999.01174.x. 10027960

[14] Carruthers VB, Moreno SNJ, Sibley LD 1999 Ethanol and acetaldehyde elevate intracellular [Ca^2+^] calcium and stimulate microneme discharge in *Toxoplasma gondii*. Biochem J 342:379–386. doi:10.1042/bj3420379. 10455025 PMC1220475

[15] Lovett JL, Marchesini N, Moreno SN, Sibley LD 2002 *Toxoplasma gondii* microneme secretion involves intracellular Ca^2+^ release from IP_3_/ryanodine sensitive stores. J Biol Chem 277:25870–25876. doi:10.1074/jbc.M202553200. 12011085

[16] Gurnett AM, Liberator PA, Dulski PM, Salowe SP, Donald RG, Anderson JW, Wiltsie J, Diaz CA, Harris G, Chang B, Darkin-Rattray SJ, Nare B, Crumley T, Blum PS, Misura AS, Tamas T, Sardana MK, Yuan J, Biftu T, Schmatz DM 2002 Purification and molecular characterization of cGMP-dependent protein kinase from apicomplexan parasites. A novel chemotherapeutic target. J Biol Chem 277:15913–15922. doi:10.1074/jbc.M108393200. 11834729

[17] Donald RG, Allocco JJ, Singh SB, Nare B, Salowe SP, Wiltsie J, Liberator PA 2002 *Toxoplasma gondii* cyclic GMP-dependent kinase: chemotherapeutic targeting of an essential parasite protein kinase. Eukaryot Cell 1:317–328. doi:10.1128/EC.1.3.317-328.2002. 12455981 PMC118020

[18] Diaz CA, Allocco J, Powles MA, Yeung L, Donald RG, Anderson JW, Liberator PA 2006 Characterization of Plasmodium falciparum cGMP-dependent protein kinase (PfPKG): antiparasitic activity of a PKG inhibitor. Mol Biochem Parasitol 146:78–88. doi:10.1016/j.molbiopara.2005.10.020. 16325279

[19] Donald RGK, Liberator PA 2002 Molecular characterization of a coccidian parasite cGMP dependent protein kinase. Mol Biochem Parasitol 120:165–175. doi:10.1016/S0166-6851(01)00451-0. 11897122

[20] Shen B, Brown KM, Lee TD, Sibley LD 2014 Efficient gene disruption in diverse strains of *Toxoplasma gondii* using CRISPR/CAS9. mBio 5:e01114-14. doi:10.1128/mBio.01114-14. 24825012 PMC4030483

[21] Sidik SM, Hackett CG, Tran F, Westwood NJ, Lourido S 2014 Efficient genome engineering of Toxoplasma gondii using CRISPR/Cas9. PLoS One 9:e100450. doi:10.1371/journal.pone.0100450. 24971596 PMC4074098

[22] Sidik SM, Huet D, Ganesan SM, Huynh MH, Wang T, Nasamu AS, Thiru P, Saeij JP, Carruthers VB, Niles JC, Lourido S 2016 A genome-wide CRISPR screen in toxoplasma identifies essential apicomplexan genes. Cell 166:1423–1435.e12. doi:10.1016/j.cell.2016.08.019. 27594426 PMC5017925

[23] Nishimura K, Fukagawa T, Takisawa H, Kakimoto T, Kanemaki M 2009 An auxin-based degron system for the rapid depletion of proteins in nonplant cells. Nat Methods 6:917–922. doi:10.1038/nmeth.1401. 19915560

[24] McCoy JM, Whitehead L, van Dooren GG, Tonkin CJ 2012 TgCDPK3 regulates calcium-dependent egress of *Toxoplasma gondii* from host cells. PLoS Pathog 8:e1003066. doi:10.1371/journal.ppat.1003066. 23226109 PMC3514314

[25] Doerig C 2004 Protein kinases as targets for anti-parasitic chemotherapy. Biochim Biophys Acta 1697:155–168. doi:10.1016/j.bbapap.2003.11.021. 15023358

[26] Donald RG, Zhong T, Wiersma H, Nare B, Yao D, Lee A, Allocco J, Liberator PA 2006 Anticoccidial kinase inhibitors: identification of protein kinase targets secondary to cGMP-dependent protein kinase. Mol Biochem Parasitol 149:86–98. doi:10.1016/j.molbiopara.2006.05.003. 16765465

[27] Hopp CS, Bowyer PW, Baker DA 2012 The role of cGMP signalling in regulating life cycle progression of plasmodium. Microbes Infect 14:831–837. doi:10.1016/j.micinf.2012.04.011. 22613210 PMC3484397

[28] Radke JR, Donald RG, Eibs A, Jerome ME, Behnke MS, Liberator P, White MW 2006 Changes in the expression of human cell division autoantigen-1 influence Toxoplasma gondii growth and development. PLoS Pathog 2:e105. doi:10.1371/journal.ppat.0020105. 17069459 PMC1626100

[29] Odell AV, Tran F, Foderaro JE, Poupart S, Pathak R, Westwood NJ, Ward GE 2015 Yeast three-hybrid screen identifies TgBRADIN/GRA24 as a negative regulator of Toxoplasma gondii bradyzoite differentiation. PLoS One 10:e0120331. doi:10.1371/journal.pone.0120331. 25789621 PMC4366382

[30] Bansal A, Ojo KK, Mu J, Maly DJ, Van Voorhis WC, Miller LH 2016 Reduced activity of mutant calcium-dependent protein kinase 1 is compensated in Plasmodium falciparum through the action of protein kinase G. mBio 7:e02011-16. doi:10.1128/mBio.02011-16. 27923926 PMC5142624

[31] Wang JL, Huang SY, Behnke MS, Chen K, Shen B, Zhu XQ 2016 The past, present, and future of genetic manipulation in Toxoplasma gondii. Trends Parasitol 32:542–553. doi:10.1016/j.pt.2016.04.013. 27184069

[32] Kanemaki M, Sanchez-Diaz A, Gambus A, Labib K 2003 Functional proteomic identification of DNA replication proteins by induced proteolysis in vivo. Nature 423:720–724. doi:10.1038/nature01692. 12768207

[33] Banaszynski LA, Chen LC, Maynard-Smith LA, Ooi AG, Wandless TJ 2006 A rapid, reversible, and tunable method to regulate protein function in living cells using synthetic small molecules. Cell 126:995–1004. doi:10.1016/j.cell.2006.07.025. 16959577 PMC3290523

[34] Herm-Götz A, Agop-Nersesian C, Münter S, Grimley JS, Wandless TJ, Frischknecht F, Meissner M 2007 Rapid control of protein level in the apicomplexan Toxoplasma gondii. Nat Methods 4:1003–1005. doi:10.1038/nmeth1134. 17994029 PMC2601725

[35] Russo I, Oksman A, Vaupel B, Goldberg DE 2009 A calpain unique to alveolates is essential in Plasmodium falciparum and its knockdown reveals an involvement in pre-S-phase development. Proc Natl Acad Sci U S A 106:1554–1559. doi:10.1073/pnas.0806926106. 19164769 PMC2629787

[36] Kubota T, Nishimura K, Kanemaki MT, Donaldson AD 2013 The Elg1 replication factor C-like complex functions in PCNA unloading during DNA replication. Mol Cell 50:273–280. doi:10.1016/j.molcel.2013.02.012. 23499004

[37] Philip N, Waters AP 2015 Conditional degradation of plasmodium calcineurin reveals functions in parasite colonization of both host and vector. Cell Host Microbe 18:122–131. doi:10.1016/j.chom.2015.05.018. 26118994 PMC4509507

[38] Kreidenweiss A, Hopkins AV, Mordmüller B 2013 2A and the auxin-based degron system facilitate control of protein levels in Plasmodium falciparum. PLoS One 8:e78661. doi:10.1371/journal.pone.0078661. 24236031 PMC3827281

[39] Morawska M, Ulrich HD 2013 An expanded tool kit for the auxin-inducible degron system in budding yeast. Yeast 30:341–351. doi:10.1002/yea.2967. 23836714 PMC4171812

[40] Hopp CS, Flueck C, Solyakov L, Tobin A, Baker DA 2012 Spatiotemporal and functional characterisation of the Plasmodium falciparum cGMP-dependent protein kinase. PLoS One 7:e48206. doi:10.1371/journal.pone.0048206. 23139764 PMC3489689

[41] Lourido S, Tang K, Sibley LD 2012 Distinct signalling pathways control toxoplasma egress and host-cell invasion. EMBO J 31:4524–4534. doi:10.1038/emboj.2012.299. 23149386 PMC3545288

[42] Huynh MH, Carruthers VB 2009 Tagging of endogenous genes in a *Toxoplasma gondii* strain lacking Ku80. Eukaryot Cell 8:530–539. doi:10.1128/EC.00358-08. 19218426 PMC2669203

[43] Bastin P, Bagherzadeh A, Matthews KR, Gull K 1996 A novel epitope tag system to study protein targeting and organelle biogenesis in *Trypanosoma brucei*. Mol Biochem Parasitol 77:235–239. doi:10.1016/0166-6851(96)02598-4. 8813669

[44] Starnes GL, Coincon M, Sygusch J, Sibley LD 2009 Aldolase is essential for energy production and bridging adhesin-actin cytoskeletal interactions during parasite invasion of host cells. Cell Host Microbe 5:353–364. doi:10.1016/j.chom.2009.03.005. 19380114 PMC2683947

[45] Carruthers VB, Sherman GD, Sibley LD 2000 The toxoplasma adhesive protein MIC2 is proteolytically processed at multiple sites by two parasite-derived proteases. J Biol Chem 275:14346–14353. doi:10.1074/jbc.275.19.14346. 10799515

[46] Alaganan A, Fentress SJ, Tang K, Wang Q, Sibley LD 2014 Toxoplasma GRA7 effector increases turnover of immunity-related GTPases and contributes to acute virulence in the mouse. Proc Natl Acad Sci U S A 111:1126–1131. doi:10.1073/pnas.1313501111. 24390541 PMC3903209

[47] Frénal K, Polonais V, Marq JB, Stratmann R, Limenitakis J, Soldati-Favre D 2010 Functional dissection of the apicomplexan glideosome molecular architecture. Cell Host Microbe 8:343–357. doi:10.1016/j.chom.2010.09.002. 20951968

[48] Alexander DL, Mital J, Ward GE, Bradley PJ, Boothroyd JC 2005 Identification of the moving junction complex of *Toxoplasma gondii*: a collaboration between distinct secretory organelles. PLoS Pathog 1:e17. doi:10.1371/journal.ppat.0010017. 16244709 PMC1262624

[49] Soldati D, Boothroyd JC 1993 Transient transfection and expression in the obligate intracellular parasite *Toxoplasma gondii*. Science 260:349–352. doi:10.1126/science.8469986. 8469986

