# Supplementary figures and images for: Plasma Membrane Association by N-Acylation Governs PKG Function in Toxoplasma gondii
Source: mBio. 2017 May 2;8(3):e00375-17. doi: 10.1128/mBio.00375-17 (PMC5414004; doi:10.1128/mBio.00375-17)

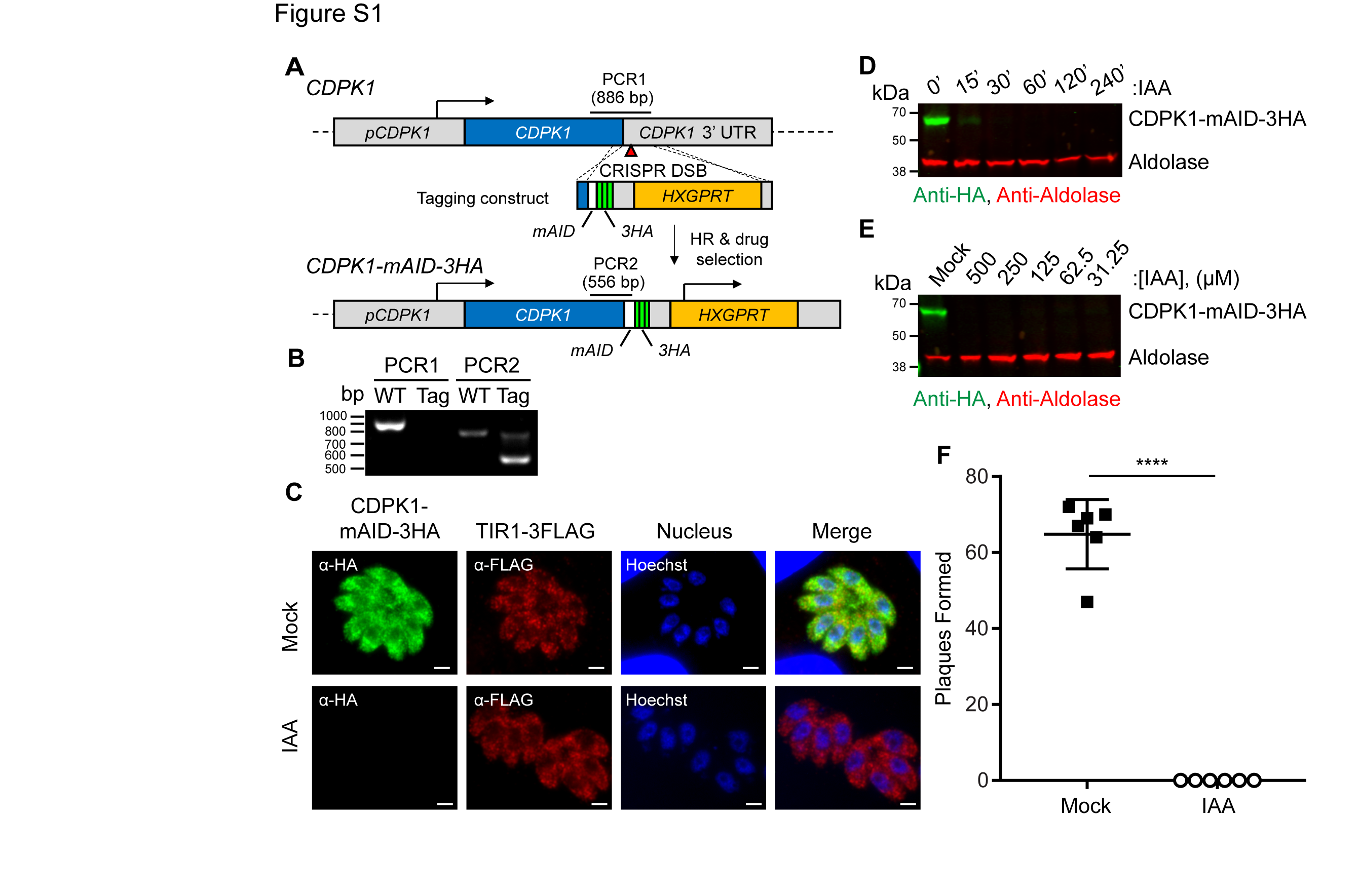

Supplement: FIG S1 [file mbo002173295sf1.tif]

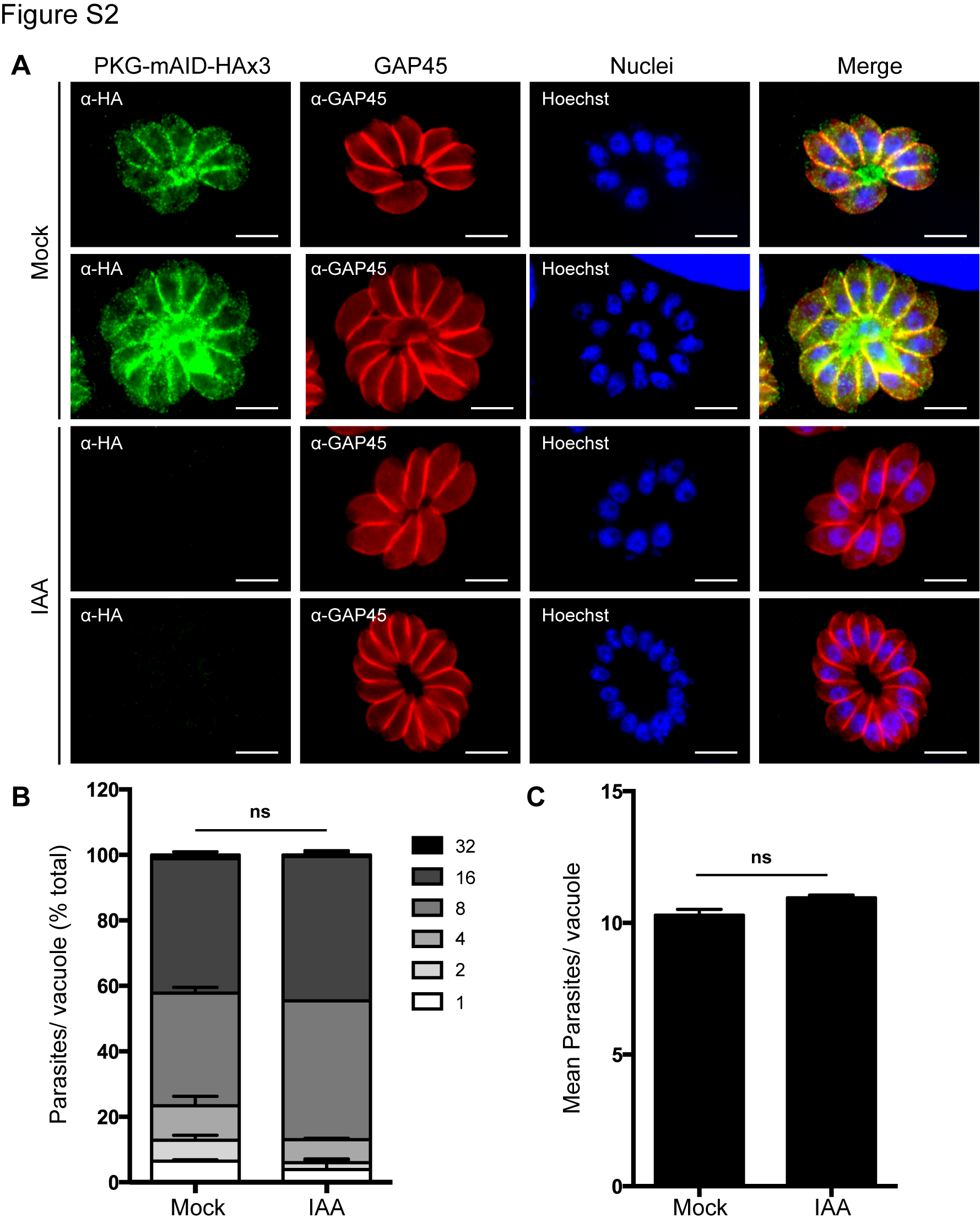

Supplement: FIG S2 [file mbo002173295sf2.tif]

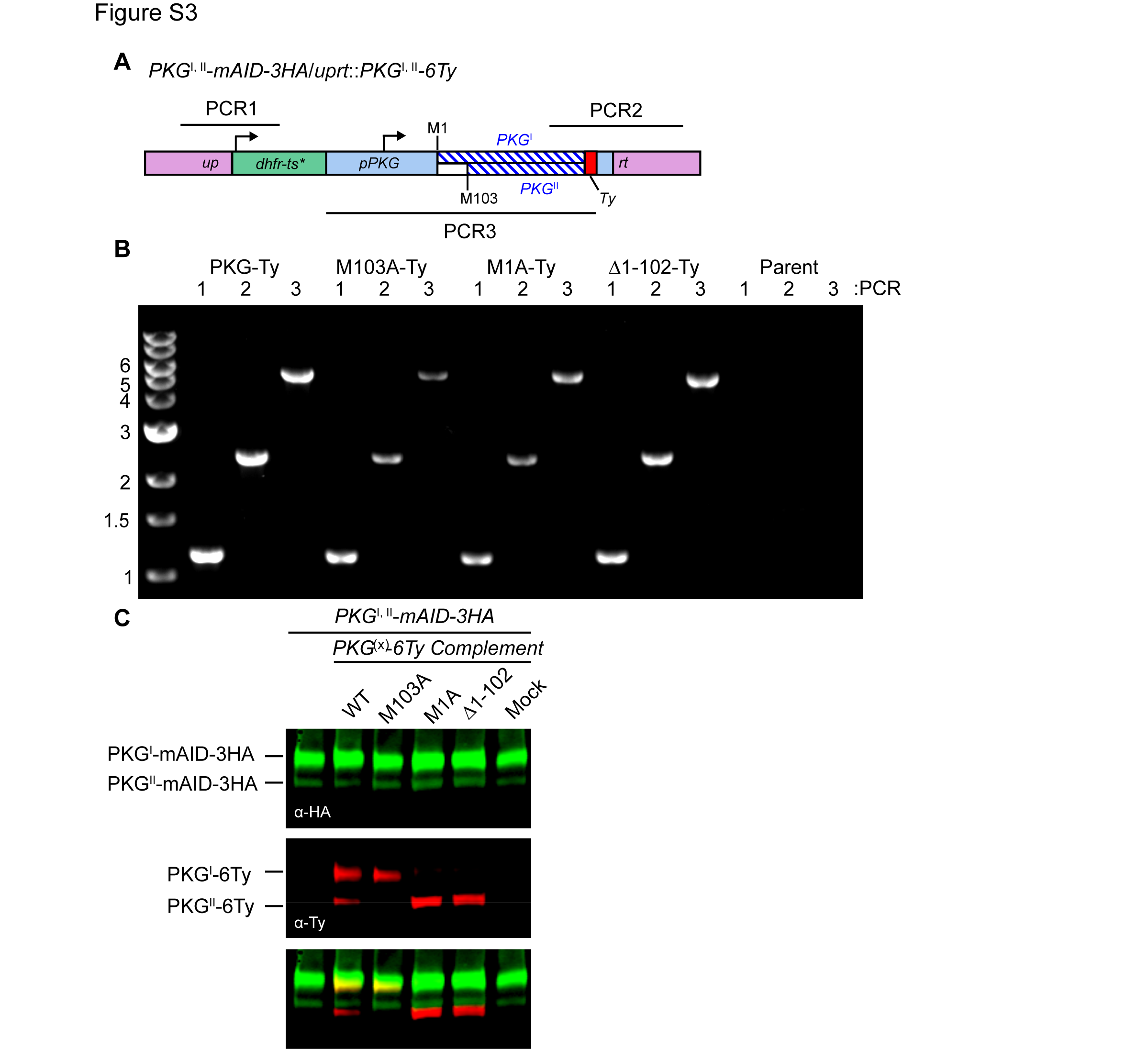

Supplement: FIG S3 [file mbo002173295sf3.tif]

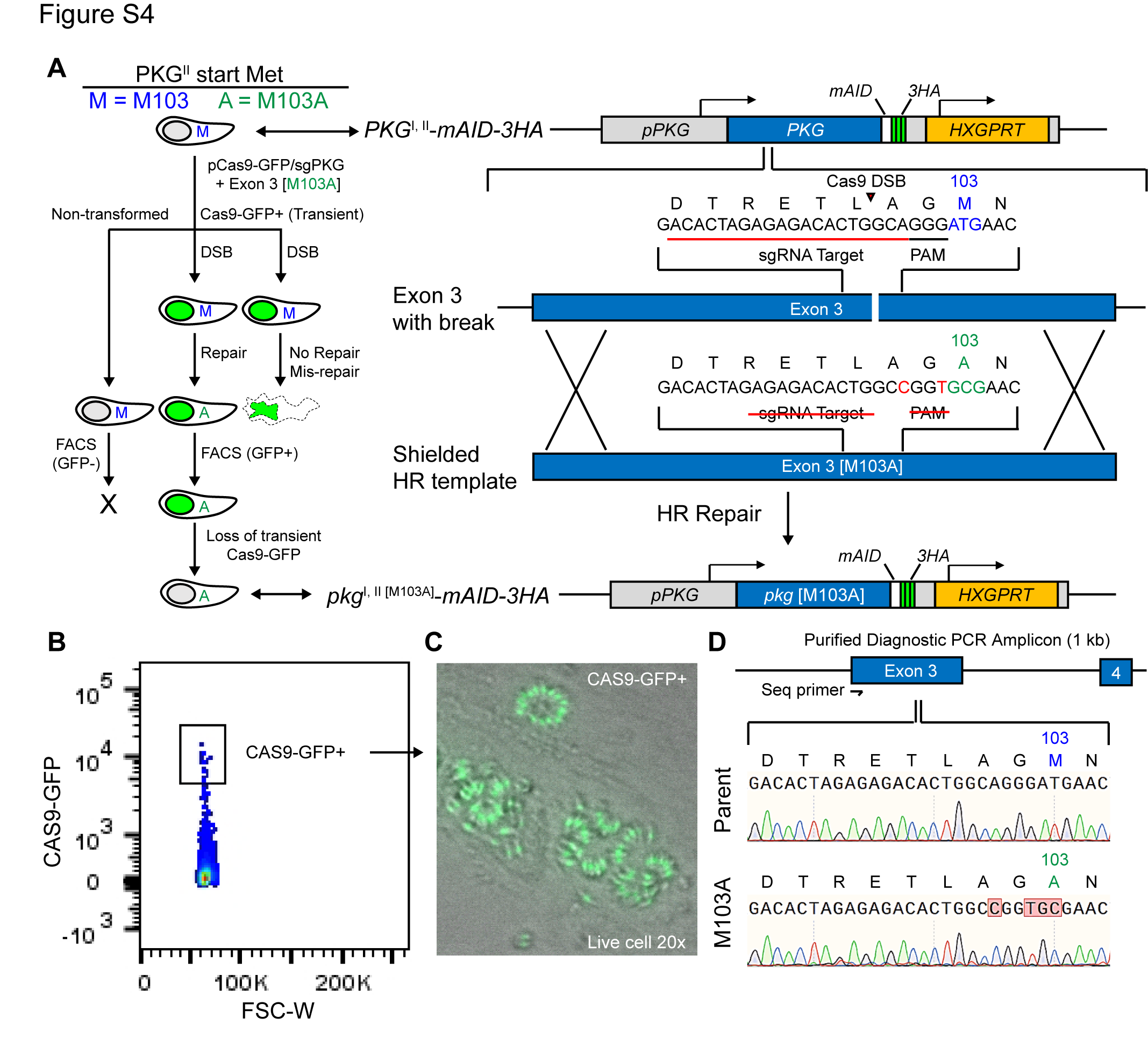

Supplement: FIG S4 [file mbo002173295sf4.tif]

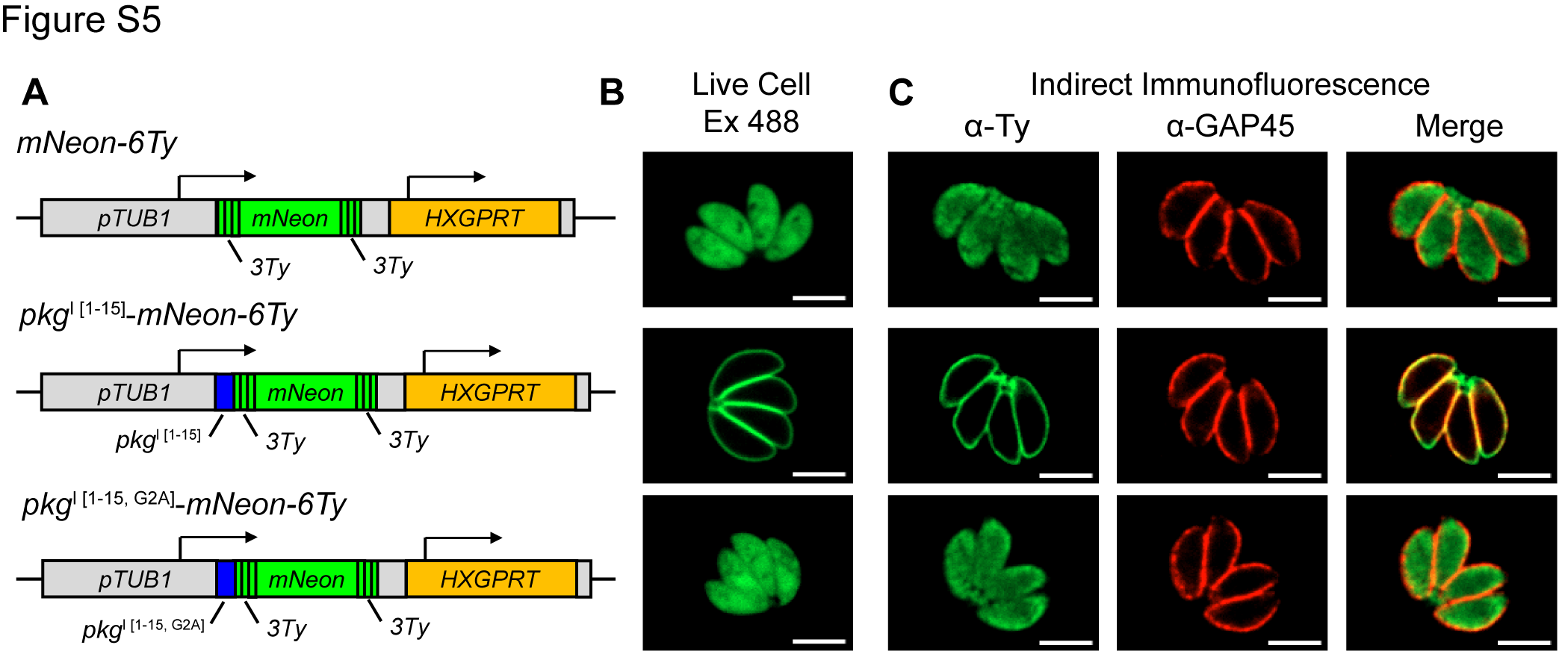

Supplement: FIG S5 [file mbo002173295sf5.tif]
