# Supplementary material for: Plasma Membrane Association by N-Acylation Governs PKG Function in Toxoplasma gondii
Source: mBio. 2017 May 2;8(3):e00375-17. doi: 10.1128/mBio.00375-17 (PMC5414004; doi:10.1128/mBio.00375-17)
Supplement: TABLE S1 [file mbo002173295st1.docx]

**Table S1. Plasmids used in this study.**

| **#** | **Plasmid** | **Description** | **Usage** |
| --- | --- | --- | --- |
| p1 | p*TUB1*:*YFP-YFP*, *SAG1*:*CAT* | Destination vector for *TUB1*-driven transgene expression in *T. gondii* with a CAT drug selectable marker | Subcloning |
| p2 | p*TUB1*:*OsTIR1*-*3FLAG*, *SAG1*:*CAT* | TIR1-3FLAG fusion driven by a *TUB1* promoter with a CAT drug selectable marker. The TIR1 CDS from *Oryza sativa* was codon optimized for *T. gondii* expression | Stable TIR1 expression |
| p3 | p*TUB1*:*YFP*, *DHFR-TS*:*HXGPRT* | Destination vector for *TUB1*-driven transgene expression in *T. gondii* with an HXGPRT drug selectable marker | Subcloning |
| p4 | p*TUB1*:*YFP*-*mAID*-*3HA*, *DHFR-TS*:*HXGPRT* | YFP-mAID-3HA fusion driven by a minimal *TUB1* promoter with an HXGPRT drug selectable marker. The mAID CDS from *Arabidopsis thaliana* auxin-responsive protein IAA17^E66-S133^ was codon optimized for *T. gondii* expression | Reporter for auxin-induced protein degradation in *T. gondii*; PCR template for generating *CDPK1* and *PKG* mAID-3HA tagging amplicons. |
| p5 | p*SAG1*:*CAS9-GFP*, *U6*:sg*UPRT* | *Streptococcus pyogenes* CAS9 fused to GFP driven by a *SAG1* promoter and CRISPR sgRNA targeting *UPRT* (GGCGTCTCGATTGTGAGAGC) driven from a Pol III *U6* promoter. | CRISPR plasmid targeting *UPRT*; PCR template for generating *CDPK1-* and *PKG*-targeting CRISPR plasmids by site-directed mutagenesis. |
| p6 | p*SAG1*:*CAS9-GFP*, *U6*:sg*CDPK1* | *Streptococcus pyogenes* CAS9 fused to GFP driven by a *SAG1* promoter and CRISPR sgRNA targeting *CDPK1* 3’ UTR (GAGAATAGACGTCACGCACA) driven from a Pol III *U6* promoter. | CRISPR plasmid targeting *CDPK1* 3’ UTR for C-terminal tagging |
| p7 | p*SAG1*:*CAS9-GFP*, *U6*:sg*PKG* | *Streptococcus pyogenes* CAS9 fused to GFP driven by a *SAG1* promoter and CRISPR sgRNA targeting *PKG* 3’ UTR (AGCGGCACTTTCAGCACTGA) driven from a Pol III *U6* promoter. | CRISPR plasmid targeting *PKG* 3’ UTR for C-terminal tagging |
| p8 | p*TUB1*:*CAT* | Destination vector with a CAT drug selectable marker | Subcloning |
| p9 | p*TUB1*:*CAT*, *PKG*^I, II^*-Ty* | *PKG*^I, II^*-Ty* fusion with a CAT drug selectable marker | Subcloning; PCR template for generating *pkg-Ty* mutant plasmids by site-directed mutagenesis. |
| p10 | p*TUB1*:*CAT*, *pkg*^I [M103A]^*-Ty* | *pkg*^I [M103A]^*-Ty* fusion with a CAT drug selectable marker | Subcloning |
| p11 | p*TUB1*:*CAT*, *pkg*^II [M1A]^*-Ty* | *pkg^II^* ^[M1A]^*-Ty* fusion with a CAT drug selectable marker | Subcloning |
| p12 | p*TUB1*:*CAT*, *pkg*^II [Δ1-102]^*-Ty* | *pkg^II^* ^[Δ1-102]^*-Ty* fusion with a CAT drug selectable marker | Subcloning |
| p13 | p*UPRT*::*dhfr-ts*^[S36R, T83N]^ | *dhfr-ts*^[S36R, T83N]^ drug selectable marker flanked by homology arms from *UPRT* close to the sg*UPRT* targeting region. Destination vector for PKG complementation constructs. | *UPRT* disruption; Mock PKG complementation; Subcloning |
| p14 | p*UPRT*::*dhfr-ts*^[S36R, T83N]^, *PKG*^I, II^*-Ty* | *PKG*^I, II^*-Ty* fusion with *dhfr-ts*^[S36R, T83N]^ drug selectable marker flanked by homology arms from *UPRT* close to the sg*UPRT* targeting region. | *PKG-mAID-3HA* complementation |
| p15 | p*UPRT*::*dhfr-ts*^[S36R, T83N]^, *pkg*^I [M103A]^*-Ty* | *pkg*^I [M103A]^*-Ty* fusion with *dhfr-ts*^[S36R, T83N]^ drug selectable marker flanked by homology arms from *UPRT* close to the sg*UPRT* targeting region. | *PKG-mAID-3HA* complementation |
| p16 | p*UPRT*::*dhfr-ts*^[S36R, T83N]^, *pkg*^II [M1A]^*-Ty* | *pkg^II^* ^[M1A]^*-Ty* fusion with *dhfr-ts*^[S36R, T83N]^ drug selectable marker flanked by homology arms from *UPRT* close to the sg*UPRT* targeting region. | *PKG-mAID-3HA* complementation |
| p17 | p*UPRT*::*dhfr-ts*^[S36R, T83N]^, *pkg*^II^ ^[Δ1-102]^*-Ty* | *pkg^II^* ^[Δ1-102]^*-Ty* fusion with *dhfr-ts*^[S36R, T83N]^ drug selectable marker flanked by homology arms from *UPRT* close to the sg*UPRT* targeting region. | *PKG-mAID-3HA* complementation |
| p18 | p*UPRT*::*dhfr-ts*^[S36R, T83N]^, *PKG*^I, II^*-6Ty* | *PKG*^I, II^*-6Ty* fusion with *dhfr-ts*^[S36R, T83N]^ drug selectable marker flanked by homology arms from *UPRT* close to the sg*UPRT* targeting region. | *PKG-mAID-3HA* complementation |
| p19 | p*UPRT*::*dhfr-ts*^[S36R, T83N]^, *pkg*^I [M103A]^*-6Ty* | *pkg*^I [M103A]^*-6Ty* fusion with *dhfr-ts*^[S36R, T83N]^ drug selectable marker flanked by homology arms from *UPRT* close to the sg*UPRT* targeting region. | *PKG-mAID-3HA* complementation |
| p20 | p*UPRT*::*dhfr-ts*^[S36R, T83N]^, *pkg*^II [M1A]^*-6Ty* | *pkg^II^* ^[M1A]^*-6Ty* fusion with *dhfr-ts*^[S36R, T83N]^ drug selectable marker flanked by homology arms from *UPRT* close to the sg*UPRT* targeting region. | *PKG-mAID-3HA* complementation |
| p21 | p*UPRT*::*dhfr-ts*^[S36R, T83N]^, *pkg*^II [Δ1-102]^*-6Ty* | *pkg^II^* ^[Δ1-102]^*-6Ty* fusion with *dhfr-ts*^[S36R, T83N]^ drug selectable marker flanked by homology arms from *UPRT* close to the sg*UPRT* targeting region. | *PKG-mAID-3HA* complementation |
| p22 | p*SAG1*:*CAS9-GFP*, *U6*:sg*PKG*[M103] | *Streptococcus pyogenes* CAS9 fused to GFP driven by a *SAG1* promoter and CRISPR sgRNA targeting *PKG* near M103 (ACACTAGAGAGACACTGGCA) driven from a Pol III *U6* promoter. | CRISPR plasmid targeting endogenous *PKG* near M103 to generate pkg^I [M103A]^ mutant. |
| p23 | p*TUB1*:*CAT*, *pkg*^I [M103A]^*-Ty*_v2 | *pkg*^I [M103A]^*-Ty* fusion containing shielding silent mutations to resist CRISPR sgPKG[M103] with a CAT drug selectable marker | PCR template for generating  *pkg*^[M103A]^ 252 bp homology repair amplicon for markerless genome editing strategy used to generate pkg^I [M103A]^ mutant |
| p24 | p*6Ty*, *DHFR-TS*:*HXGPRT* | 6Ty-tagging vector with an HXGPRT drug selectable marker | PCR template for generating *6Ty* amplicon with Gibson flanks to p*UPRT*::*dhfr-ts*^[S36R, T83N]^, *PKG*^I, II^ |
| p25 | p*TUB1*:*mNeon-6Ty*, *DHFR-TS*:*HXGPRT* | *mNeon-Ty* fusion driven by a *TUB1* promoter with an HXGPRT drug selectable marker | Stable mNeon-6Ty expression |
| p26 | p*TUB1*:*pkg*^I [1-15]^*-mNeon-6Ty*, *DHFR-TS*:*HXGPRT* | *pkg^I [1-15]^-mNeon-Ty* fusion driven by a *TUB1* promoter with an HXGPRT drug selectable marker | Stable pkg^I [1-15]^-mNeon-6Ty expression |
| p27 | p*TUB1*:*pkg*^I [1-15, G2A]^*-mNeon-6Ty*, *DHFR-TS*:*HXGPRT* | *pkg^I [1-15, G2A]^-mNeon-6Ty* fusion driven by a *TUB1* promoter with an HXGPRT drug selectable marker | Stable pkg^I [1-15, G2A]^-mNeon-6Ty expression |
| p28 | p*UPRT*::*dhfr-ts*^[S36R, T83N]^, *pkg*^I [1-15]^*-pkg*^II [104-994]^*-6Ty* | *pkg*^I [1-15]^*-pkg*^II [104-994]^*-6Ty* fusion with *dhfr-ts*^[S36R, T83N]^ drug selectable marker flanked by homology arms from *UPRT* close to the sg*UPRT* targeting region. | *PKG-mAID-3HA* complementation |
| p29 | p*UPRT*::*dhfr-ts*^[S36R, T83N]^, *cdpk3*^[1-15]^*-pkg*^II [104-994]^*-6Ty* | *cdpk3* ^[1-15]^*-pkg*^II [104-994]^*-6Ty* fusion with *dhfr-ts*^[S36R, T83N]^ drug selectable marker flanked by homology arms from *UPRT* close to the sg*UPRT* targeting region. | *PKG-mAID-3HA* complementation |
