# Supplementary material for: Plasma Membrane Association by N-Acylation Governs PKG Function in Toxoplasma gondii
Source: mBio. 2017 May 2;8(3):e00375-17. doi: 10.1128/mBio.00375-17 (PMC5414004; doi:10.1128/mBio.00375-17)
Supplement: TABLE S2 [file mbo002173295st2.docx]

**Table S2 - Primers used in this study.**

| **#** | **Primer name** | **5’ – 3’ sequence** | **Usage** | **Template DNA** | **Final plasmid** |
| --- | --- | --- | --- | --- | --- |
| P1 | pTUB1:_, CAT Gib F | GCATGCCACAGAAGCTG | Amplify vector backbone from p*TUB1*:*YFP-YFP*, *SAG1*:*CAT* | p1 | p2 |
| P2 | pTUB1:_, CAT Gib R | TTTAGATCTAAAAGGGAATTCAAGAAAAAATGC |  |  |  |
| P3 | pTUB1:_, HXGPRT Gib F | CCCGGGCATATGTAGAAAAGTTG | Amplify vector backbone from p*TUB1*:YFP, *DHFR-TS*:*HXGPRT* | p3 | p4 |
| P4 | pTUB1:_, HXGPRT Gib R | AGATCTAAAAGGGAATTCAAGAA |  |  |  |
| P5 | pU6 R | AACTTGACATCCCCATTTAC | Universal reverse primer for mutating the sgRNA targeting sequence of p*SAG1*:*CAS9-GFP*, *U6*:sg*UPRT* | p5 | p6, p7, p23 |
| P6 | pU6-sgCDPK1 F | GAGAATAGACGTCACGCACAGTTTTAGAGCTAGAAATAGC | Forward primer for mutating p*SAG1*:*CAS9-GFP*, *U6*:sg*UPRT* to p*SAG1*:*CAS9-GFP*, *U6*:sg*CDPK1* | p5 | p6 |
| P7 | M13 R | CAGGAAACAGCTATGAC | Sequencing primer to confirm p*SAG1*:*CAS9-GFP*, *U6*:sg[target] sequences | p6, p7, p23 | N/A |
| P8 | CDPK1-mAID F | TTGACGAGTTTCAACAGATGCTCTTGAAGCTCTGCGGAAACgctagcAAGGGCTCGG | Amplify *CDPK1-mAID-3HA*, *DHFR*-*TS*:*HXGPRT* tagging amplicon from p*TUB1*:*YFP*-*mAID*-*3HA*, *DHFR-TS*:*HXGPRT* | p4 | N/A |
| P9 | CDPK1-mAID R | AGAGGGGTGCACGGGAGTCTGGGGGAGAATAGACGTCACGCAATACGACTCACTATAGG |  |  |  |
| P10 | CDPK1 3’ F | CGGATGTTTGACTCCGACAACT | Forward primer for CDPK1 Diagnostic PCRs 1,2 | gDNA | N/A |
| P11 | CDPK1 3’ R | GGCTTAGGCTAGGTCTCTCTTTG | Reverse primer for CDPK1 Diagnostic PCR 1 | gDNA | N/A |
| P12 | mAID R | GATCTTTAGGACACGCGCTC | Reverse primer for CDPK1 and PKG Diagnostic PCR 2 | gDNA | N/A |
| P13 | pU6-sgPKG F | AGCGGCACTTTCAGCACTGAGTTTTAGAGCTAGAAATAGC | Forward primer for mutating p*SAG1*:*CAS9-GFP*, *U6*:sg*UPRT* to p*SAG1*:*CAS9-GFP*, *U6*:sg*PKG* | p5 | p7 |
| P14 | PKG-mAID F | GATCGTTTTGGAAGACGAGTATGACTGGGACAAGGATTTCGCTAGCAAGGGCTCGGGC | Amplify *PKG-mAID-3HA*, *DHFR*-*TS*:*HXGPRT* tagging amplicon from p*TUB1*:*YFP*-*mAID*-*3HA*, *DHFR-TS*:*HXGPRT* | p4 | N/A |
| P15 | PKG-mAID R | GCACTTTCAGCACTGACGGGGAACAAACACCTAAGCTGAAATAGGGCGAATTGGAGCTCC |  |  |  |
| P16 | PKG 3’ F | GCATTTAGCCGCATACACAC | Forward primer for *PKG* Diagnostic PCRs 1,2 | gDNA | N/A |
| P17 | PKG 3’ R | GACTCCTTAGGTTGCACAGA | Reverse primer for *PKG* Diagnostic PCR 1 | gDNA | N/A |
| P18 | PKG 5’ UTR Gib F | TTCTTGCGAGCTCGGTACCCCTTCCTTCTCTTTGCGTGCC | Amplify *PKG* 5’ UTR from RH gDNA with 5’ Gibson flank to SmaI-linearized p*TUB1*:*CAT* | gDNA | p9 |
| P19 | PKG 5’ UTR Gib R | TTTCGCGCGAGGAGAGACAA |  |  |  |
| P20 | PKG-Ty Gib F | TTGTCTCTCCTCGCGCGAAAATGGGCGCTTGCATTTCCAA | Amplify *PKG* coding sequence from RH cDNA with 5’ Gibson flank to *PKG* 5’ UTR and 3’ Ty tag | cDNA |  |
| P21 | PKG-Ty Gib R | TCAATCGAGCGGGTCCTGGTTCGTGTGGACCTCGAAATCCTTGTCCCAGTCAT |  |  |  |
| P22 | PKG 3’ UTR Gib F | ACCAGGACCCGCTCGATTGATTTTTCAGCTTAGGTGTTTGTTC | Amplify *PKG* 3’ UTR from RH gDNA with 5’ Gibson flank to Ty tag and 3’ Gibson flank to SmaI-linearized p*TUB1*:*CAT* | gDNA |  |
| P23 | PKG 3’ UTR Gib R | GTCGACTCTAGAGGATCCCCAACTGAGGAAACGAGTAGGA |  |  |  |
| P24 | PKG Seq 1 F | ACTCGGTTCATCTCGAT | Sanger sequencing primers for *PKG* plasmids | p9-12, p14-21, p23, p28, p29 | N/A |
| P25 | PKG Seq 2 F | TCTGACTGGGAACTGAC |  |  |  |
| P26 | PKG Seq 3 F | TCACCTTCAAGAAAGGC |  |  |  |
| P27 | PKG Seq 4 F | GACTACGTCATTCTGGA |  |  |  |
| P28 | PKG Seq 5 F | CAAGTGGTCAGAGTGGT |  |  |  |
| P29 | PKG Seq 6 F | ACCTGATGAAGAGGCTT |  |  |  |
| P30 | PKG M103A F | ACTGGCAGGGGCGAACTCTCCC | Mutate p*TUB1*:*CAT*, *PKG*^I, II^*-Ty* to p*TUB1*:*CAT*, *pkg*^I [M103A]^*-Ty* | p9 | p10 |
| P31 | PKG M103A R | GTCTCTCTAGTGTCTTTCTTC |  |  |  |
| P32 | PKG M1A F | TCGCGCGAAAGCGGGCGCTTGC | Mutate p*TUB1*:*CAT*, *PKG*^I, II^*-Ty* to p*TUB1*:*CAT*, *pkg*^II [M1A]^*-Ty* | p9 | p11 |
| P33 | PKG M1A R | GGAGAGACAACGAGGACC |  |  |  |
| P34 | PKG Δ1-102 F | ATGAACTCTCCCAAGACTCTG | Mutate p*TUB1*:*CAT*, *PKG*^I, II^*-Ty* to p*TUB1*:*CAT*, *pkg*^II [Δ1-102]^*-Ty* | p9 | p12 |
| P35 | PKG Δ1-102 F | TTTCGCGCGAGGAGAGAC |  |  |  |
| P36 | pUPRT::DHFR F | CTTTCTTGTACAAAGTGGCG | PCR-linearize p*UPRT*::*dhfr-ts*^[S36R, T83N]^ vector for subcloning | p13 | p14-17 |
| P37 | pUPRT::DHFR R | CTGGGTAGGAATTCATCCTG |  |  |  |
| P38 | pUPRT::DHFR, PKG Gib F | CAGGATGAATTCCTACCCAGCTTCCTTCTCTTTGCGTGCC | Amplify *PKG* (5’ UTR through 3’ UTR) from p*TUB1*:*CAT*, *PKG*^I, II^*-Ty* (or mutant derivatives) with Gibson flanks to p*UPRT*::*dhfr-ts*^[S36R, T83N]^ | p9  p10  p11  p12 | p14  p15  p16  p17 |
| P39 | pUPRT::DHFR, PKG Gib R | CGCCACTTTGTACAAGAAAGAACTGAGGAAACGAGTAGGA |  |  |  |
| P40 | upUPRT F | TGCATCTTCGTAGAGGTAAACAG | *uprt*::*dhfr-ts*^[S36R, T83N]^, *PKG-Ty* Diagnostic PCR 1 (5’ Integration) | gDNA | N/A |
| P41 | inDHFR R | TACCAGTCATGGACGAGATCG |  |  |  |
| P42 | inPKG 3’ F | TGAGAACGTTCCGCGACA | *uprt*::*dhfr-ts*^[S36R, T83N]^, *PKG-Ty* Diagnostic PCR 2 (3’ Integration) | gDNA | N/A |
| P43 | dnUPRT R | GCTACTAACAAGTGACGACG |  |  |  |
| P44 | inPKG F | CTTCCTTCTCTTTGCGTGCC | *uprt*::*dhfr-ts*^[S36R, T83N]^, *PKG-Ty* Diagnostic PCR 3 (Internal) | gDNA | N/A |
| P45 | inTy R | TCAATCGAGCGGGTCCTGGTTCGTGTGG |  |  |  |
| P46 | PKG-6Ty F | TGATTTTTCAGCTTAGGTGTTTGTTC | Amplify p*UPRT*::*dhfr-ts*^[S36R, T83N]^, *PKG*^I, II^*-Ty* (or mutant derivatives) to swap *Ty* for *6Ty* | p14  p15  p16  p17 | p18  p19  p20  p21 |
| P47 | PKG-6Ty R | CAGCTGGGTCGAGCCCGA |  |  |  |
| P48 | 6Ty F | GAAGTCCACACGAATCAGGACCC | Amplify 6Ty sequence with Gibson flanks to p*UPRT*::*dhfr-ts*^[S36R, T83N]^, *PKG*^I, II^ | p24 | p18-21 |
| P49 | 6Ty R | GTCGAGCGGGTCCTGGTTTGTA |  |  |  |
| P50 | pU6-sgPKG[M103] F | ACACTAGAGAGACACTGGCAGTTTTAGAGCTAGAAATAGC | Forward primer for mutating p*SAG1*:*CAS9-GFP*, *U6*:sg*UPRT* to p*SAG1*:*CAS9-GFP*, *U6*:sg*PKG*[M103] | p5 | p22 |
| P51 | PKG M103A_v2 F | ACTGGCCGGTGCGAACTCTCCCAAGACT | Forward primer for use with M103A R to mutate p*TUB1*:*CAT*, *PKG*^I, II^*-Ty* to p*TUB1*:*CAT*, *pkg*^I [M103A]^*-Ty*_v2 | p9 | p23 |
| P52 | PKG Exon 3 F | GGTAGACGGAACGCGAGCCT | Amplify *pkg* exon 3 markerless genome editing amplicon from p*TUB1*:*CAT*, *pkg*^I [M103A]^*-Ty*_v2; PKG Exon 3 F also used as sequencing primer to confirm M103A mutation in pkg^I [M103A]^-mAID-3HA parasites | p23 | N/A |
| P53 | PKG Exon 3 R | GGTTTTTCTTTCGGGCCTTCTGGA |  |  |  |
| P54 | PKG Intron 2 F | CACAGCGAGTTGAGACCTGC | Amplify locus surrounding PKG Exon 3 for purification and sequencing to confirm M103A mutation in pkg^I [M103A]^-mAID-3HA parasites | gDNA | N/A |
| P55 | PKG Exon 4 R | GTAGAGAATCCGCTTACGTTGAG |  |  |  |
| P56 | pDHFR:HXGPRT F | CCCGGGCATATGTAGAAAAGTTG | Amplify vector backbone from p*TUB1*:*YFP*-*mAID*-*3HA*, *DHFR-TS*:*HXGPRT* excluding *TUB1:YFP-mAID-3HA* sequence | p4 | p25 |
| P57 | pDHFR:HXGPRT R | GGGCCCGAATTCCCGTCCT |  |  |  |
| P58 | TUB1 F | GAGGACGGGAATTCGGGCCCTAACGACACAAGGAGATGCG | Amplify *TUB1* promoter from p*TUB1*:*YFP-YFP*, *SAG1*:*CAT* with 5’ Gibson flank to p*DHFR-*TS:*HXGPRT* vector backbone and 3’ *mNeon-6Ty* gBlock. | p1 | p25 |
| P59 | TUB1 R | CGAGCCCGAGCCCTTGCTAGCCATTTTAGATCTAAAAGGGAATTCAAG |  |  |  |
| P60 | PKG 1-15 F | AGTTCGGCTCGCGTTTCACGGGCTAGCAAGGGCTCGGGC | Mutate p*TUB1*:*mNeon-6Ty*, *DHFR-TS*:*HXGPRT* to p*TUB1*:*pkg*^I [1-15]^*-mNeon-6Ty*, *DHFR-TS*:*HXGPRT* | p25 | p26 |
| P61 | PKG 1-15 R | ATTTTTGGAAATGCAAGCGCCCATTTTAGATCTAAAAGGGAATTCAAGAAAAAATGCC |  |  |  |
| P62 | PKG 1-15 G2A R | ATTTTTGGAAATGCAAGCTGCCATTTTAGATCTAAAAGGGAATTCAAGAAAAAATGCC | With PKG 1-15 F, mutate p*TUB1*:*mNeon-6Ty*, *DHFR-TS*:*HXGPRT* to p*TUB1*:*pkg*^I [1-15, G2A]^*-mNeon-6Ty*, *DHFR-TS*:*HXGPRT* | p25 | p27 |
| P63 | PKG Δ16-103 F | AACTCTCCCAAGACTCTGGAG | Mutate p*UPRT*::*dhfr-ts*^[S36R, T83N]^, *pkg*^II [Δ1-102]^*-6Ty* to p*UPRT*::*dhfr-ts*^[S36R, T83N]^, *pkg*^I [1-15]^*-pkg*^II [104-994]^*-6Ty* | p21 | p28 |
| P64 | PKG Δ16-103 R | CCGTGAAACGCGAGCCGAA |  |  |  |
| P65 | CDPK3-PKG F | CCCCACTCCAAGCATGCAGGCAACTCTCCCAAGACTCTGGA | Mutate p*UPRT*::*dhfr-ts*^[S36R, T83N]^, *pkg*^II [Δ1-102]^*-6Ty* to p*UPRT*::*dhfr-ts*^[S36R, T83N]^, *cdpk3*^[1-15]^*-pkg*^II [104-994]^*-6Ty* | p21 | p29 |
| P66 | CDPK3-PKG R | ATTCTTGGAGTGGACGCACCCCATTTTCGCGCGAGGAGAGA |  |  |  |
