# Supplementary material for: Plasma Membrane Association by N-Acylation Governs PKG Function in Toxoplasma gondii
Source: mBio. 2017 May 2;8(3):e00375-17. doi: 10.1128/mBio.00375-17 (PMC5414004; doi:10.1128/mBio.00375-17)
Supplement: TEXT S1 [file mbo002173295s1.docx]

**Text S1 – Plasmid construction**

All plasmids (p#) and primers (P#) used in this study are listed with descriptions and usages in Appendix Tables S1 and S2, respectively. Codon optimization was performed with an online tool https://www.idtdna.com/CodonOpt.

**Auxin-inducible degron plasmid construction.**

The plasmid p*TUB1*:*YFP-YFP*, *SAG1*:*CAT* (p1) was made previously (called tubYFP-YFP/sagCAT) (1). The plasmid p*TUB1*:*OsTIR1*-*3FLAG*, *SAG1*:*CAT* (p2) was made by two fragment Gibson assembly: Fragment 1: PCR-truncated (P1, P2) vector backbone derived from p*TUB1*:*YFP-YFP*, *SAG1*:*CAT* (p1) where *YFP-YFP* is excluded; Fragment 2: gBlock® of *Oryza sativa* *TIR1* CDS (codon-optimized for expression in *T. gondii*) in frame with *3FLAG*. The plasmid p*TUB1*:*YFP*-*mAID*-*3HA*, *DHFR-TS*:*HXGPRT* (p4) was made by two fragment Gibson assembly: Fragment 1: PCR-linearized (P3, P4) vector backbone from p*TUB1*:*YFP*, *DHFR-TS*:*HXGPRT* (p3); Fragment 2: gBlock® of mAID from *Arabidopsis thaliana* auxin-responsive protein IAA17^E66-S133^ (codon-optimized for *T. gondii* expression) in frame with *3HA*. The mAID amino acid sequence is based on AtIAA17^K65-K132^ “mini-AID” first defined in *Saccharomyces cerevisiae* (2).

**Ectopic PKG plasmid construction.**

The plasmid p*TUB1*:*CAT* (p8) was made by cloning the *TUB1*:*CAT* selectable marker from pT/230 (called TUB1/CAT) (3) into pUC19 at KasI and SacI sites. The plasmid p*TUB1*:*CAT*, *PKG*^I, II^*-Ty* (p9) was made by four fragment Gibson assembly: Fragment 1: SmaI-linearized p*TUB1*:*CAT* (p8); Fragment 2: PCR-amplified (P18, P19) *PKG* promoter (2304 bp) from RH gDNA; Fragment 3: PCR-amplified (P20, P21) coding sequence of *PKG* (2982 bp + *Ty*) from RH cDNA; Fragment 4: PCR-amplified (P22, P23) *PKG* 3’ UTR (629 bp) from RH gDNA. The plasmid p*TUB1*:*CAT*, *PKG*^I, II^*-Ty* (p9) was used as a template for site-directed mutagenesis PCRs to yield p*TUB1*:*CAT*, *PKG*^I [M103A]^*-Ty* (p10; P30, P31), p*TUB1*:*CAT*, *PKG*^II [M1A]^*-Ty* (p11; P32, P33), p*TUB1*:*CAT*, *PKG*^II [Δ1-102]^*-Ty* (p12; P34, P35), and p*TUB1*:*CAT*, *PKG*^I [M103A]^*-Ty*_v2(p23; P51, P31). The plasmid p*UPRT*::*dhfr-ts*^[S36R, T83N]^ (p13) was made previously (called pUPRT::DHFR-I) (4). The plasmids p*UPRT*::*dhfr-ts*^[S36R, T83N]^, *PKG*^I, II^*-Ty* (p14), p*UPRT*::*dhfr-ts*^[S36R, T83N]^, *pkg*^I [M103A]^*-Ty* (p15), p*UPRT*::*dhfr-ts*^[S36R, T83N]^, *pkg*^II^ ^[M1A]^*-Ty* (p16), and p*UPRT*::*dhfr-ts*^[S36R, T83N]^, *pkg*^II^ ^[Δ1-102]^*-Ty* (p17) were made by PCR-amplifying (P38, P39) the PKG-Ty cassettes from p9-p12 and assembling them into PCR-linearized (P36, P37) p*UPRT*::*dhfr-ts*^[S36R, T83N]^ (p13). Additionally, 6Ty versions of these plasmids were also constructed using two fragment Gibson assembly where *Ty* was removed by PCR (P46, P47) and replaced with a *6Ty* PCR amplicon (P48, P49) from p*6Ty*, *DHFR-TS:HXGPRT* (p24) yielding p*UPRT*::*dhfr-ts*^[S36R, T83N]^, *PKG*^I, II^*-6Ty* (p18), p*UPRT*::*dhfr-ts*^[S36R, T83N]^, *pkg*^I [M103A]^*-6Ty* (p19), p*UPRT*::*dhfr-ts*^[S36R, T83N]^, *pkg*^II [M1A]^*-6Ty* (p20), p*UPRT*::*dhfr-ts*^[S36R, T83N]^, *pkg*^II^ ^[Δ1-102]^*-6Ty* (p21), p*UPRT*::*dhfr-ts*^[S36R, T83N]^. The plasmid p*UPRT*::*dhfr-ts*^[S36R, T83N]^, *pkg*^II^ ^[Δ1-102]^*-6Ty* (p21) was used as a template for site-directed mutagenesis PCRs to yield *pkg*^I [1-15]^*-pkg*^II [104-994]^*-6Ty* (p28; P63, P64) and p*UPRT*::*dhfr-ts*^[S36R, T83N]^, *cdpk3*^[1-15]^*-pkg*^II [104-994]^*-6Ty* (p29; P65, P66). The plasmid p*TUB1*:*mNeon-6Ty*, *DHFR-TS*:*HXGPRT* (p25) was made by three fragment Gibson assembly: Fragment 1: PCR-truncated (P56, P57) vector backbone from p*TUB1*:*YFP*-*mAID*-*3HA*, *DHFR-TS*:*HXGPRT* (p4) where *TUB1*:*YFP-mAID-3HA* is excluded; Fragment 2: PCR-amplified (P58, P59) *TUB1* promoter from p*TUB1*:*YFP-YFP*, *SAG1*:*CAT* (p1). Fragment 3: gBlock® of mNeonGreen (5) CDS (codon-optimized for expression in *T. gondii*) flanked by *3Ty* sequences. The plasmid p*TUB1*:*mNeon-6Ty*, *DHFR-TS*:*HXGPRT* (p25) was used as a template for site-directed mutagenesis PCRs yielding p*TUB1*:*pkg*^I [1-15, G2A]^*-mNeon-6Ty*, *DHFR-TS*:*HXGPRT* (p25; P60, P61) and p*TUB1*:*pkg*^I [1-15, G2A]^*-mNeon-6Ty*, *DHFR-TS*:*HXGPRT* (p26; P60, P62).

**Cas9 plasmid construction**

All CRISPR/Cas9 plasmids used in this study were derived from p*SAG1*:*CAS9-GFP*, *U6*:sg*UPRT* (p5) by site-directed mutagenesis to alter the 20 nt targeting sequence as described previously (4). They include: p*SAG1*:*CAS9-GFP*, *U6*:sg*CDPK1* (p6; P5, P6); p*SAG1*:*CAS9-GFP*, *U6*:sg*PKG* (p7; P5, P13); p*SAG1*:*CAS9-GFP*, *U6*:sg*PKG*[M103] (p22; P5, P50).

**References**

1. **Gubbels MJ, Li C, Striepen B.** 2003. High-throughput growth assay for *Toxoplasma gondii* using yellow fluorescent protein. Antimicrob Agents Chem **47:**309-316.

2. **Kubota T, Nishimura K, Kanemaki MT, Donaldson AD.** 2013. The Elg1 replication factor C-like complex functions in PCNA unloading during DNA replication. Mol Cell **50:**273-280.

3. **Nagel SD, Boothroyd JC.** 1988. The alpha- and beta-tubulins of *Toxoplasma gondii* are encoded by single copy genes containing multiple introns. Molecular and Biochemical Parasitology **29:**261-273.

4. **Shen B, Brown KM, Lee TD, Sibley LD.** 2014. Efficient gene disruption in diverse strains of *Toxoplasma gondii* using CRISPR/CAS9. mBio **13;5(3):e01114-14**.

5. **Shaner NC, Lambert GG, Chammas A, Ni Y, Cranfill PJ, Baird MA, Sell BR, Allen JR, Day RN, Israelsson M, Davidson MW, Wang J.** 2013. A bright monomeric green fluorescent protein derived from Branchiostoma lanceolatum. Nat Methods **10:**407-409.
