# Supplementary material for: Plasma Membrane Association by N-Acylation Governs PKG Function in Toxoplasma gondii
Source: mBio. 2017 May 2;8(3):e00375-17. doi: 10.1128/mBio.00375-17 (PMC5414004; doi:10.1128/mBio.00375-17)
Supplement: TEXT S2 [file mbo002173295s2.docx]

**Appendix**

Proposed genetic nomenclature guidelines for *Toxoplasma gondii.*

Kevin M. Brown^1^, Shaojun Long^1^, and L. David Sibley^1^

*^1^ Department of Molecular Microbiology, Washington University School of Medicine, 660 S. Euclid Ave., St. Louis, MO, 63110*

The absence of a modern standardized genetic nomenclature system for use in *T. gondii* prompted us to develop these guidelines for naming wild-type and mutant genes, proteins, genotypes, and parasite lines.

Wild-type nomenclature

|  | Single species investigated | Multiple species investigated | *T. gondii* lineage | Multiple isoforms from single gene^1^ | Fusions (N-, C-) |
| --- | --- | --- | --- | --- | --- |
| Gene symbol guidelines | Italicized uppercase letters and numbers | Italicized uppercase genus letter, lowercase species letter | Subscripted roman numerals | Superscripted roman numerals | Hyphen |
| Example 1 | *CDPK1* | *TgCDPK1, NcCDPK1* | *CDPK1*_I_, *CDPK1*_II_, etc. | N/A | *HA-CDPK1* |
| Example 2 | *PKG* | *TgPKG*, *PfPKG* | *PKG*_I_, *PKG*_II_, etc. | *PKG*^I, II^ | *PKG*^I, II^*-HA* |
| Protein symbol guidelines | Uppercase letters and numbers | Uppercase genus letter, lowercase species letter | Subscripted roman numerals | Superscripted roman numerals | Hyphen |
| Example 1 | CDPK1 | TgCDPK1, NcCDPK1 | CDPK1_I_, CDPK1_II_, etc. | N/A | HA-CDPK1 |
| Example 2 | PKG | TgPKG, PfPKG | PKG_I_, PKG_II_, etc. | PKG^I^, PKG^II^ | PKG^I^-HA, PKG^II^-HA |
| ^1^ The gene *TgPKG* (ToxoDB: TgGT1_311360) encodes two protein isoforms, PKG^I (M1-F994)^ and PKG^II (M103-F994)^, based on alternative translation initiation sites. Isoform designations may be added to the gene and protein symbols when necessary to distinguish the isoforms from one another. | | | | | |

Mutant nomenclature

|  | Point mutant | Truncation^2^ | Deletion | Replacement | Disruption | Complement or Merodiploid |
| --- | --- | --- | --- | --- | --- | --- |
| Mutant gene symbol guidelines | Italicized lowercase letters and numbers, superscripted isoform and [mutation] | Italicized lowercase letters and numbers, superscripted isoform and [mutation] | Delta symbol, italicized lowercase letters and numbers, superscripted isoform | Delta symbol, italicized lowercase letters and numbers, double colon, transgene | Italicized lowercase letters and numbers, double colon, transgene | Endogenous gene/ectopic gene |
| Example 1 | *cdpk1*^[G128M]^ | *cdpk1*^[Δ2-20]^ | Δ*cdpk1* | Δ*cdpk1*::*CAT* | *cdpk1*::*CAT* | Δ*cdpk1*/*CDPK1-Ty* |
| Example 2 | *pkg*^I, II [M103A]^ | *pkg*^I, II [Δ1-102]^ | Δ*pkg*^I, II^ | Δ*pkg*^I, II^::*CAT* | *pkg*^I, II^::*CAT* | Δ*pkg*^I, II^/*PKG*^I, II^*-Ty* |
| Mutant protein symbol guidelines | Lowercase letters and numbers, superscripted isoform and [mutation] | Lowercase letters and numbers, superscripted isoform and [mutation] | Lowercase letters and numbers, superscripted isoform and [mutation] | Lowercase letters and numbers, superscripted isoform and [mutation] | Lowercase letters and numbers, superscripted isoform and [mutation] | Endogenous protein/ectopic protein |
| Example 1 | cdpk1^[G128M]^ | cdpk1^[Δ2-20]^ | cdpk1^[KO]^ | cdpk1^[KO]^ | Variable | cdpk1^[KO]^/CDPK1-Ty |
| Example 2 | pkg^I [M103A]^,  pkg^II [KO]^ | pkg^I [KO]^,  pkg^II^ ^[103-994]^ | pkg^I [KO]^,  pkg^II [KO]^ | pkg^I [KO]^,  pkg^II [KO]^ | Variable | pkg^I [KO]^, pkg^II [KO]^/PKG^I^-Ty, PKG^II^-Ty |
| ^2^ The mutant gene *pkg*^I, II [Δ1-102]^ does not produce PKG^I^ but still produces PKG^II^ protein. In this case the expressed protein isoform should be written as pkg^II^ ^[103-994]^ instead of pkg^II^ ^[Δ1-102]^. | | | | | | |

Genotype nomenclature

Dedicated symbols

| Deletion | Replacement | Disruption | Fusion | Promoter | Allele | Linked genetic element | Unlinked genetic element | Merodiploid (endogenous copy/ ectopic copy) |
| --- | --- | --- | --- | --- | --- | --- | --- | --- |
| Δ | Δ :: | :: | - | : | [ ] | , | ; | / |

Example genotype

RHΔ*ku80*Δ*hxgprt*; *TUB1*:*TIR1-3FLAG, SAG1*:*CAT*; *CDPK1*-*mAID-3HA*, *DHFR-TS*:*HXGPRT*/*uprt*::*dhfr-ts*^[S36R, T83N]^, *CDPK1-6Ty*

RHΔ*hxgprt*Δ*ku80*

RH isolate with *HXGPRT* and *KU80* deletions.

*TUB1*:*TIR1-3FLAG, SAG1*:*CAT*

TIR1-3FLAG expression driven by *TUB1* promoter linked to a *CAT* drug selectable marker driven by a *SAG1* promoter.

*CDPK1*-*mAID-3HA*, *DHFR-TS*:*HXGPRT*

Endogenous *CDPK1* tagged with *mAID-3HA* linked to an *HXGPRT* drug selectable marker driven by a *DHFR-TS* promoter.

*CDPK1*-*mAID-3HA*, *DHFR-TS*:*HXGPRT*/*uprt*::*dhfr-ts*^[S36R, T83N]^, *CDPK1-6Ty*

Endogenous *CDPK1* tagged with *mAID-3HA* linked to an *HXGPRT* drug selectable marker driven by a *DHFR-TS* promoter and complemented at the *UPRT* locus with *CDPK1-6Ty* linked to a *dhfr- ts*^[S36R, T83N]^ drug selectable marker.

Notes:

1. Genotypes should begin with the isolate name followed by pertinent genetic elements in order of generation or introduction.
2. Transgenes expressed from heterologous (non-native) promoters require the “promoter:gene” designation.
3. Transgenes derived from organisms other than *T. gondii* may be denoted in the genotype using the *Genus species* two letter prefix (e.g. *OsTIR1*; *Oryza sativa TIR1*). Alternatively, this information may be included in the methods section.
4. Synthetic transgenes that have been re-coded or codon optimized for expression in *T. gondii* should be described using a genotype footnote or in the methods section.
5. Genetic fusions that do not perturb the wild-type function of the gene’s product should follow the wild-type nomenclature guidelines (e.g. *CDPK1-Ty* instead of *cdpk1-Ty*).
6. Ectopic second copy transgenes should be written with the endogenous/ectopic merodiploid convention to distinguish it from the endogenous copy. For instance, a second copy of *CDPK1* fused to *Ty* should be written as *CDPK1*/*CDPK1-Ty*. This is to avoid confusion with *CDPK1-Ty*, which would indicate that endogenous *CDPK1* was tagged with *Ty*.

Strain nomenclature

| Genotype | Shorthand strain name |
| --- | --- |
| RH | RH |
| RHΔ*hxgprt*Δ*ku80* | ku80^[KO]^ |
| RHΔ*hxgprt*Δ*ku80*; *TUB1*:*TIR1-3FLAG*, *SAG1*:*CAT* | TIR1-3FLAG |
| RHΔ*hxgprt*Δ*ku80*; *TUB1*:*TIR1-3FLAG, SAG1*:*CAT*; *CDPK1*-*mAID-3HA*, *DHFR-TS*:*HXGPRT* | CDPK1-mAID-3HA |
| RHΔ*hxgprt*Δ*ku80*; *TUB1*:*TIR1-3FLAG, SAG1*:*CAT*; *CDPK1*-*mAID-3HA*, *DHFR-TS*:*HXGPRT*/*uprt*::*dhfr-ts*^[S36R, T83N]^, *CDPK1-6Ty* | CDPK1-mAID-3HA/CDPK1-6Ty |
| RHΔ*hxgprt*Δ*ku80*; *TUB8*:*TATi*; *T7S1*:*CDPK1*/*CDPK1-6Ty* | CDPK1-iKD/CDPK1-6Ty |

Notes:

1. Strains with elaborate genotype names may be given shorthand names based on pertinent phenotypes or proteins expressed.
2. It is not necessary to denote the parental isolate when naming derivative strains unless similar derivatives were generated from independent parental isolates.
