# Supplementary material for: Plasma Membrane Association by N-Acylation Governs PKG Function in Toxoplasma gondii
Source: mBio. 2017 May 2;8(3):e00375-17. doi: 10.1128/mBio.00375-17 (PMC5414004; doi:10.1128/mBio.00375-17)
Supplement: TABLE S3 [file mbo002173295st3.docx]

**Table S3. Strains used in this study.**

| **Shorthand Strain Name** | **PKG Isoforms Expressed** | **Genotype^1^** | **CRISPR plasmids^2^** | **Associated plasmids^2, 3^** |
| --- | --- | --- | --- | --- |
| RH | PKG^I^, PKG^II^ | RH | N/A | N/A |
| ku80^[KO]^ | PKG^I^, PKG^II^ | RHΔ*hxgprt*Δ*ku80* | N/A | N/A |
| TIR1-3FLAG | PKG^I^, PKG^II^ | RHΔ*hxgprt*Δ*ku80*; *TUB1*:*TIR1-3FLAG, SAG1*:*CAT* | N/A | p2 |
| YFP-mAID-3HA | PKG^I^, PKG^II^ | RHΔ*hxgprt*Δ*ku80*; *TUB1*:*TIR1-3FLAG, SAG1*:*CAT*; *TUB1*:*YFP-mAID-3HA*, *DHFR-TS*:*HXGPRT* | N/A | p2 |
|  |  |  | N/A | p4 |
| CDPK1-mAID-3HA | PKG^I^, PKG^II^ | RHΔ*hxgprt*Δ*ku80*; *TUB1*:*TIR1-3FLAG, SAG1*:*CAT*; *CDPK1*-*mAID-3HA*, *DHFR-TS*:*HXGPRT* | N/A | p2 |
|  |  |  | p6 | p4 |
| PKG^I, II^-mAID-3HA | PKG^I^-mAID-3HA, PKG^II^-mAID-3HA | RHΔ*hxgprt*Δ*ku80*; *TUB1*:*TIR1-3FLAG, SAG1*:*CAT*; *PKG*^I, II^-*mAID-3HA*, *DHFR-TS*:*HXGPRT* | N/A | p2 |
|  |  |  | p7 | p4 |
| PKG^I, II^-mAID-3HA/mock | PKG^I^-mAID-3HA, PKG^II^-mAID-3HA | RHΔ*hxgprt*Δ*ku80*; *TUB1*:*TIR1-3FLAG, SAG1*:*CAT*; *PKG*^I, II^-*mAID-3HA*, *DHFR-TS*:*HXGPRT*; *uprt*::*dhfr-ts*^[S36R, T83N]^ | N/A | p2 |
|  |  |  | p7 | p4 |
|  |  |  | p5 | p13 |
| PKG^I, II^-Ty | PKG^I^-mAID-3HA, PKG^II^-mAID-3HA/  PKG^I^-Ty, PKG^II^-Ty | RHΔ*hxgprt*Δ*ku80*; *TUB1*:*TIR1-3FLAG, SAG1*:*CAT*; *PKG*^I, II^-*mAID-3HA*, *DHFR-TS*:*HXGPRT*/*uprt*::*dhfr-ts*^[S36R, T83N]^, *PKG*^I/II^*-Ty* | N/A | p2 |
|  |  |  | p7 | p4 |
|  |  |  | p5 | p14 |
| pkg^I [M103A]^-Ty | PKG^I^-mAID-3HA, PKG^II^-mAID-3HA/  pkg^I [M103A]^-Ty | RHΔ*hxgprt*Δ*ku80*; *TUB1*:*TIR1-3FLAG, SAG1*:*CAT*; *PKG*^I, II^-*mAID-3HA*, *DHFR-TS*:*HXGPRT*/*uprt*::*dhfr-ts*^[S36R, T83N]^, *pkg*^I, II [M103A]^*-Ty* | N/A | p2 |
|  |  |  | p7 | p4 |
|  |  |  | p5 | p15 |
| pkg^II [M1A]^-Ty | PKG^I^-mAID-3HA, PKG^II^-mAID-3HA/  pkg^II [M1A]^-Ty | RHΔ*hxgprt*Δ*ku80*; *TUB1*:*TIR1-3FLAG, SAG1*:*CAT*; *PKG*^I, II^-*mAID-3HA*, *DHFR-TS*:*HXGPRT*/*uprt*::*dhfr-ts*^[S36R, T83N]^, *pkg*^I, II [M1A]^*-Ty* | N/A | p2 |
|  |  |  | p7 | p4 |
|  |  |  | p5 | p16 |
| pkg^II [103-994]^-Ty | PKG^I^-mAID-3HA, PKG^II^-mAID-3HA/  pkg^II [103-994]^-Ty | RHΔ*hxgprt*Δ*ku80*; *TUB1*:*TIR1-3FLAG, SAG1*:*CAT*; *PKG*^I, II^-*mAID-3HA*, *DHFR-TS*:*HXGPRT*/*uprt*::*dhfr-ts*^[S36R, T83N]^, *pkg*^I, II [Δ1-102]^*-Ty* | N/A | p2 |
|  |  |  | p7 | p4 |
|  |  |  | p5 | p17 |
| PKG^I, II^-6Ty | PKG^I^-mAID-3HA, PKG^II^-mAID-3HA/  PKG^I^-6Ty, PKG^II^-6Ty | RHΔ*hxgprt*Δ*ku80*; *TUB1*:*TIR1-3FLAG, SAG1*:*CAT*; *PKG*^I, II^-*mAID-3HA*, *DHFR-TS*:*HXGPRT*/*uprt*::*dhfr-ts*^[S36R, T83N]^, *PKG*^I/II^*-6Ty* | N/A | p2 |
|  |  |  | p7 | p4 |
|  |  |  | p5 | p18 |
| pkg^I [M103A]^-6Ty | PKG^I^-mAID-3HA, PKG^II^-mAID-3HA/  pkg^I [M103A]^-6Ty | RHΔ*hxgprt*Δ*ku80*; *TUB1*:*TIR1-3FLAG, SAG1*:*CAT*; *PKG*^I, II^-*mAID-3HA*, *DHFR-TS*:*HXGPRT*/*uprt*::*dhfr-ts*^[S36R, T83N]^, *pkg*^I, II [M103A]^*-6Ty* | N/A | p2 |
|  |  |  | p7 | p4 |
|  |  |  | p5 | p19 |
| pkg^II [M1A]^-6Ty | PKG^I^-mAID-3HA, PKG^II^-mAID-3HA/  pkg^II [M1A]^-6Ty | RHΔ*hxgprt*Δ*ku80*; *TUB1*:*TIR1-3FLAG, SAG1*:*CAT*; *PKG*^I, II^-*mAID-3HA*, *DHFR-TS*:*HXGPRT*/*uprt*::*dhfr-ts*^[S36R, T83N]^, *pkg*^I, II [M1A]^*-6Ty* | N/A | p2 |
|  |  |  | p7 | p4 |
|  |  |  | p5 | p20 |
| pkg^II [103-994]^-6Ty | PKG^I^-mAID-3HA, PKG^II^-mAID-3HA/  pkg^II [103-994]^-6Ty | RHΔ*hxgprt*Δ*ku80*; *TUB1*:*TIR1-3FLAG, SAG1*:*CAT*; *PKG*^I, II^-*mAID-3HA*, *DHFR-TS*:*HXGPRT*/*uprt*::*dhfr-ts*^[S36R, T83N]^, *pkg*^I, II [Δ1-102]^*-6Ty* | N/A | p2 |
|  |  |  | p7 | p4 |
|  |  |  | p5 | p21 |
| pkg^I [M103A]^-mAID-3HA | pkg^I [M103A]^-mAID-3HA | RHΔ*hxgprt*Δ*ku80*; *TUB1*:*TIR1-3FLAG, SAG1*:*CAT*; *pkg*^I [M103A]^-*mAID-3HA*, *DHFR-TS*:*HXGPRT* | N/A | p2 |
|  |  |  | p7 | p4 |
|  |  |  | p22 | p23 |
| mNeon-6Ty | PKG^I^, PKG^II^ | RHΔ*hxgprt*; *TUB1*:*3Ty-mNeon-3Ty*, *DHFR-TS*:*HXGPRT* | N/A | p25 |
| pkg^I^ ^[1-15]^-mNeon-6Ty | PKG^I^, PKG^II^, pkg^I^ ^[1-15]^-mNeon-6Ty | RHΔ*hxgprt*; *TUB1*:*pkg*^I [1-15]^*-3Ty-mNeon-3Ty*, *DHFR-TS*:*HXGPRT* | N/A | p26 |
| pkg^I [1-15, G2A]^-mNeon-6Ty | PKG^I^, PKG^II^, pkg^I [1-15, G2A]^-mNeon-6Ty | RHΔ*hxgprt*; *TUB1*:*pkg*^I [1-15, G2A]^*-3Ty-mNeon-3Ty*, *DHFR-TS*:*HXGPRT* | N/A | p27 |
| pkg^I [1-15]^-pkg^II [104-994]^-6Ty | PKG^I^-mAID-3HA, PKG^II^-mAID-3HA/  pkg^I [1-15]^-pkg^II [104-994]^-6Ty | RHΔ*hxgprt*Δ*ku80*; *TUB1*:*TIR1-3FLAG, SAG1*:*CAT*; *PKG*^I, II^-*mAID-3HA*, *DHFR-TS*:*HXGPRT*/*uprt*::*dhfr-ts*^[S36R, T83N]^, *pkg*^I [1-15]^*-pkg*^II [104-994]^*-6Ty* | N/A | p2 |
|  |  |  | p7 | p4 |
|  |  |  | p5 | p28 |
| cdpk3^[1-15]^-pkg^II [104-994]^-6Ty | PKG^I^-mAID-3HA, PKG^II^-mAID-3HA/  cdpk3^[1-15]^-pkg^II [104-994]^-6Ty | RHΔ*hxgprt*Δ*ku80*; *TUB1*:*TIR1-3FLAG, SAG1*:*CAT*; *PKG*^I, II^-*mAID-3HA*, *DHFR-TS*:*HXGPRT*/*uprt*::*dhfr-ts*^[S36R, T83N]^, *cdpk3* ^[1-15]^*-pkg*^II [104-994]^*-6Ty* | N/A | p2 |
|  |  |  | p7 | p4 |
|  |  |  | p5 | p29 |

^1^ See Appendix for proposed genetic nomenclature guidelines for *Toxoplasma gondii*.

^2^ See Table S1 for plasmid descriptions.

^3^ Plasmids or plasmid-derived PCR amplicons (for tagging or gene editing) used to generate the strain.
